# Oncogenic Herpesvirus KSHV Hijacks BMP-Smad1-Id Signaling to Promote Tumorigenesis

**DOI:** 10.1371/journal.ppat.1004253

**Published:** 2014-07-10

**Authors:** Deguang Liang, Hao Hu, Shasha Li, Jiazhen Dong, Xing Wang, Yuhan Wang, Li He, Zhiheng He, Yuan Gao, Shou-Jiang Gao, Ke Lan

**Affiliations:** 1 Key Laboratory of Molecular Virology and Immunology, Institut Pasteur of Shanghai, Chinese Academy of Sciences, Shanghai, China; 2 Department of Molecular Microbiology and Immunology, Keck School of Medicine, University of Southern California, Los Angeles, California, United States of America; Wistar Institute, United States of America

## Abstract

Kaposi's sarcoma-associated herpesvirus (KSHV) is the etiological agent of Kaposi's sarcoma (KS), a malignancy commonly found in AIDS patients. Whether KS is a true neoplasm or hyperplasia has been a subject of intensive debate until recently when KSHV is unequivocally shown to efficiently infect, immortalize and transform rat primary mesenchymal precursor cells (MM). Moreover, KSHV-transformed MM cells (KMM) efficiently induce tumors with hallmark features of KS when inoculated into nude mice. Here, we showed Smad1 as a novel binding protein of KSHV latency-associated nuclear antigen (LANA). LANA interacted with and sustained BMP-activated p-Smad1 in the nucleus and enhanced its loading on the *Id* promoters. As a result, Ids were significantly up-regulated in KMM cells and abundantly expressed in human KS lesions. Strikingly, genetic and chemical inhibition of the BMP-Smad1-Id pathway blocked the oncogenic phenotype of KSHV-transformed cells *in vitro* and *in vivo*. These findings illustrate a novel mechanism by which a tumor virus hijacks and converts a developmental pathway into an indispensable oncogenic pathway for tumorigenesis. Importantly, our results demonstrate the efficacy of targeting the BMP-Smad1-Id pathway for inhibiting the growth of KSHV-induced tumors, and therefore identify the BMP pathway as a promising therapeutic target for KS.

## Author Summary

Although KSHV exerts multiple mechanisms to promote cell survival by repressing TGF-β signaling, little is known whether KSHV manipulates BMP signaling and contributes to the pathogenesis of KSHV-induced malignancies. In the present study, we have identified Smad1 as a novel binding protein of LANA by tandem affinity purification. We demonstrated that LANA up-regulated Id transcription through BMP-Smad1-Id signaling pathway. Id proteins were significantly up-regulated in KSHV-transformed MM (KMM) cells, and were abundantly expressed in human KS lesions; therefore, they were probably relevant to the development of KS. Importantly, we have shown that Ids are required to maintain the oncogenic phenotype of KMM cells *in vitro* and *in vivo*. These findings illustrate a novel mechanism by which a tumor virus hijacks and converts a developmental pathway into an indispensable oncogenic pathway for tumorigenesis. Furthermore, we showed that BMP signaling inhibitors dramatically hampered the tumorigenicity of KMM cells *in vitro* and *in vivo*. Our results demonstrate that small inhibitors targeting BMP-Smad1-Id signaling pathway are promising candidates for the treatment of KS.

## Introduction

Kaposi's sarcoma-associated herpesvirus (KSHV) is the etiological agent of Kaposi's sarcoma (KS), which is the most common malignancy in AIDS patients [1]. The KSHV-infected proliferating spindle cells are the driving force of KS [2]. KSHV mainly displays latency in spindle cells. Viral latent genes were reported to promote cell proliferation and inhibit apoptosis through various mechanisms. In particular, latency-associated nuclear antigen (LANA), a multifunctional major viral latent protein, is responsible for maintaining viral episome, inhibiting viral reactivation, and promoting cell proliferation by targeting p53, pRb and GSK-3β, etc (reviewed in [3], [4]). We have also shown that LANA contributes to cell proliferation by promoting intracellular Notch (ICN) accumulation through inhibition of Sel10-mediated ICN degradation [5], [6].

Due to the lack of *in vitro* KSHV cellular transformation model and the lack of KS cell lines, the roles of KSHV-deregulated signaling pathways in KSHV-induced cellular transformation remain unclear. The recent development of a robust model of KSHV-induced cellular transformation and tumorigenesis has made this possible [7]. Specifically, KSHV can efficiently infect, immortalize and transform primary rat embryonic metanephric mesenchymal precursor (MM) cells. KSHV-transformed MM cells (KMM) efficiently induce tumors with virological and pathological features of KS. This work has paved a way for studying the intrinsic oncogenic pathways underlying the tumorigenesis driven by KSHV latent genes. Using this system, KSHV-encoded miRNAs and vCylin were recently demonstrated to play critical roles in KSHV-induced cellular transformation and tumorigenesis [8], [9].

Bone morphogenetic proteins (BMPs) belong to the transforming growth factor β (TGF-β) superfamily. BMP signaling pathways play critical roles in diverse developmental phases [10]. In recent years, BMP signaling pathways have increasingly been the focus in cancer research, since these developmental pathways are frequently disrupted in cancer [11]. BMP signaling pathways are involved in both promotion and inhibition of cancer progression depending on the context, which is similar to the TGF-β pathway [12].

Inhibitors of DNA-binding (Id) family are major downstream targets of BMP signaling, and belong to the helix-loop-helix (HLH) family of transcription factors. There are four known members of the Id family in vertebrates (called Id1, Id2, Id3 and Id4) [13]. Id proteins do not possess a basic DNA binding domain and functions as a dominant-negative regulator of basic HLH proteins [14]. Recent evidence has revealed that Id proteins, especially Id1, are able to promote cell proliferation and cell cycle progression. Moreover, up-regulation of Id1 has been found in many types of human cancers and its expression levels are also associated with advanced tumor stage. [15]. Id1 was once reported to be up-regulated in KSHV-infected endothelial cells and in KS tissues [16], however, the mechanism and implication of Id1 up-regulation remains unclear.

In this study, Smad1 was identified as a novel LANA-binding protein. LANA up-regulated Id expression through constitutively sustaining the activation of the BMP-Smad1-Id signaling pathway, and thus contributed to the oncogenicity of KMM cells *in vitro* and *in vivo.* These studies have identified a novel viral oncogenic signaling pathway, and our data indicate that small inhibitors targeting BMP-Smad1-Id signaling pathway could be promising candidates for the treatment of KS.

## Results

### LANA interacted with BMP-activated p-Smad1 in the nucleus

**Figure 1:**
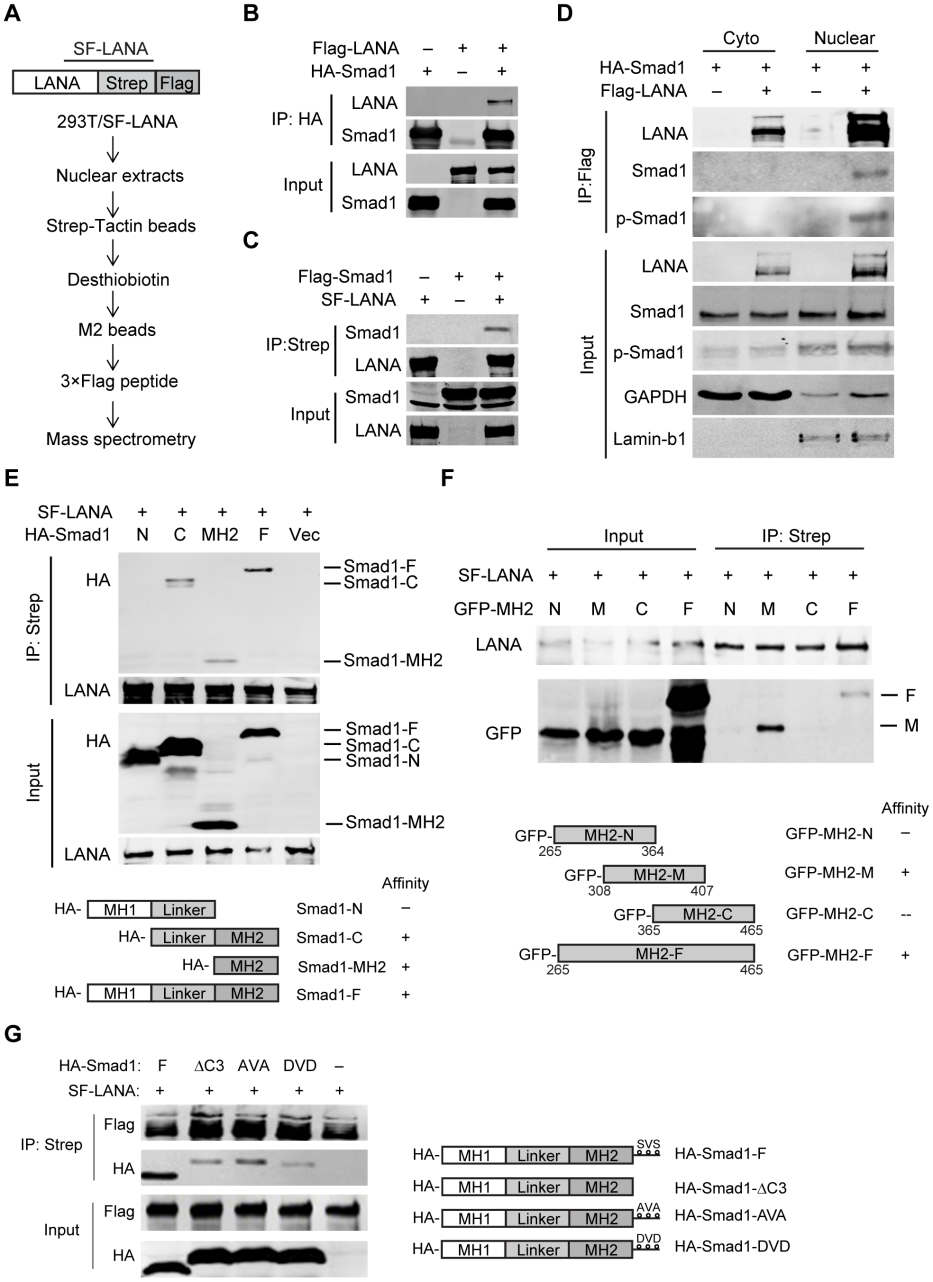
LANA interacted with BMP-activated Smad1 in the nucleus. (A) Schematic strategy showing tandem affinity purification of SF-tagged LANA Complex. (B, C) Reciprocal co-immunoprecipitation assays testing physical interactions between LANA and Smad1. Flag-LANA and HA-Smad1 (12 µg each, B), or Flag-Smad1 and SF-LANA (12 µg each, C) were co-transfected into 293T cells. Cells were lysed for co-immunoprecipitation as indicated. (D) LANA interacted with BMP-activated p-Smad1 in the nucleus. Flag-LANA and HA-Smad1 (12 µg each) were co-transfected into 293T cells. After serum starvation overnight, cells were treated with BMP2 for 2 hours, and then cells were fractionated and subjected to co-IP assay as indicated. (E) LANA interacted with Smad1 MH2 domain. Different truncated Smad1 constructs were co-transfected with SF-LANA (12 µg each) into 293T cells. Cell lysates were immunoprecipitated as indicated. (F) LANA interacted with center part of Smad1 MH2 domain. Different truncated Smad1-MH2 constructs were co-transfected with SF-LANA (12 µg each) into 293T cells. Cell lysates were immunoprecipitated as indicated. (G) LANA-Smad1 interaction did not depend on the phosphorylation of SXS motif of Smad1. HA-tagged full length Smad1 or SXS motif truncated ΔC3 mutant, or SXS motif inactivated mutant AVA, or SXS motif activated mutant DVD or vector were cotransfected with SF-LANA (12 µg each) into 293T cells. Cell lysates were immunoprecipitated as indicated.

In order to explore the novel function of LANA, we utilized Strep-Flag (SF)-tag based tandem affinity purification (SF-TAP) method to identify novel LANA-binding proteins (Fig. 1A) [17]. Smad1, a critical transducer of BMP signaling [18], was one of the hit proteins co-purified by SF-LANA [19]. We confirmed that LANA physically interacted with Smad1 in 293T cells by reciprocal co-immunoprecipitation (Co-IP) (Fig. 1B, C). We further confirmed their interaction in KSHV-infected cells (Fig. S1). LANA is predominantly located in the nucleus [20], while Smad1 shuttles from cytosol to nucleus in complex with Smad4 resulting in the transcription of BMP target genes following phosphorylation at C terminus S463/465 (SXS motif) by type I BMP receptor [18]. To determine the compartment of LANA-Smad1 interaction, 293T cells were transfected with LANA and Smad1, then treated with BMP2 and harvested for cell fraction. Co-IP assay was performed with cytoplasmic fraction and nuclear fraction respectively. As expected, LANA-Smad1 interaction was only detected in the nuclear but not in cytoplasmic fraction (Fig. 1D). Moreover, Smad1 pulled-down by LANA was recognized by a p-Smad1/5/8 antibody (Fig. 1D). Since LANA did not bind to Smad5 (Fig. S1), these results suggested that LANA interacted with BMP-activated p-Smad1 in the nucleus.

We further mapped out the Smad1-binding domain of LANA. Smad1 could be pulled down by Myc-tagged full length LANA_1–1162_ and N-terminus LANA_1–432_, but not by C-terminus LANA762–1162, negative control Intracellular Notch1 (ICN) nor control vector (Fig. S1). Therefore, N-terminus LANA_1–432_ is responsible for Smad1-binding.

Next, we mapped out the LANA-binding domain of Smad1. Smad1 has highly conserved N- and C-terminal regions known as Mad homology (MH) 1 and MH2 domains, respectively, which are linked by a linker region with a highly variable structure [18]. HA-tagged full length Smad1, Smad1-C (Linker+MH2), Smad1-MH2, but not Smad1-N (MH1+Linker) were pulled down by LANA (Fig. 1E). Therefore, Smad1 MH2 domain is responsible for LANA-binding. To narrow down the LANA-binding domain within Smad1 MH2 domain, we constructed a series of MH2 truncation mutants, termed as MH2-N, MH2-M and MH2-C respectively. Deletion of neither C-terminus of MH2 (MH2-N) nor N-terminus of MH2 (MH2-C) totally abolished its binding to LANA while the center part of MH2 (MH2-M) retained LANA binding activity (Fig. 1F). Therefore MH2-M (Smad1_308–407_) was critical for LANA-binding.

We then asked whether LANA-Smad1 interaction depended on the phosphorylation of SXS motif of Smad1. The Smad1 mutant with the SXS motif deleted (ΔC3), inactivated (AVA) or constitutively activated (DVD) [21] bound to LANA as efficiently as the wild type Smad1 (Fig. 1G). The differences of the apparent molecular weight between the wild type Smad1 and Smad1 mutants in SDS-PAGE were due to tag sizes. These results indicated that the nuclear location but not the phosphorylation of Smad1 is the restriction factor for the LANA-Smad1 interaction.

### LANA up-regulated *Id1* transcription in a BMP-Smad1 dependent manner

**Figure 2:**
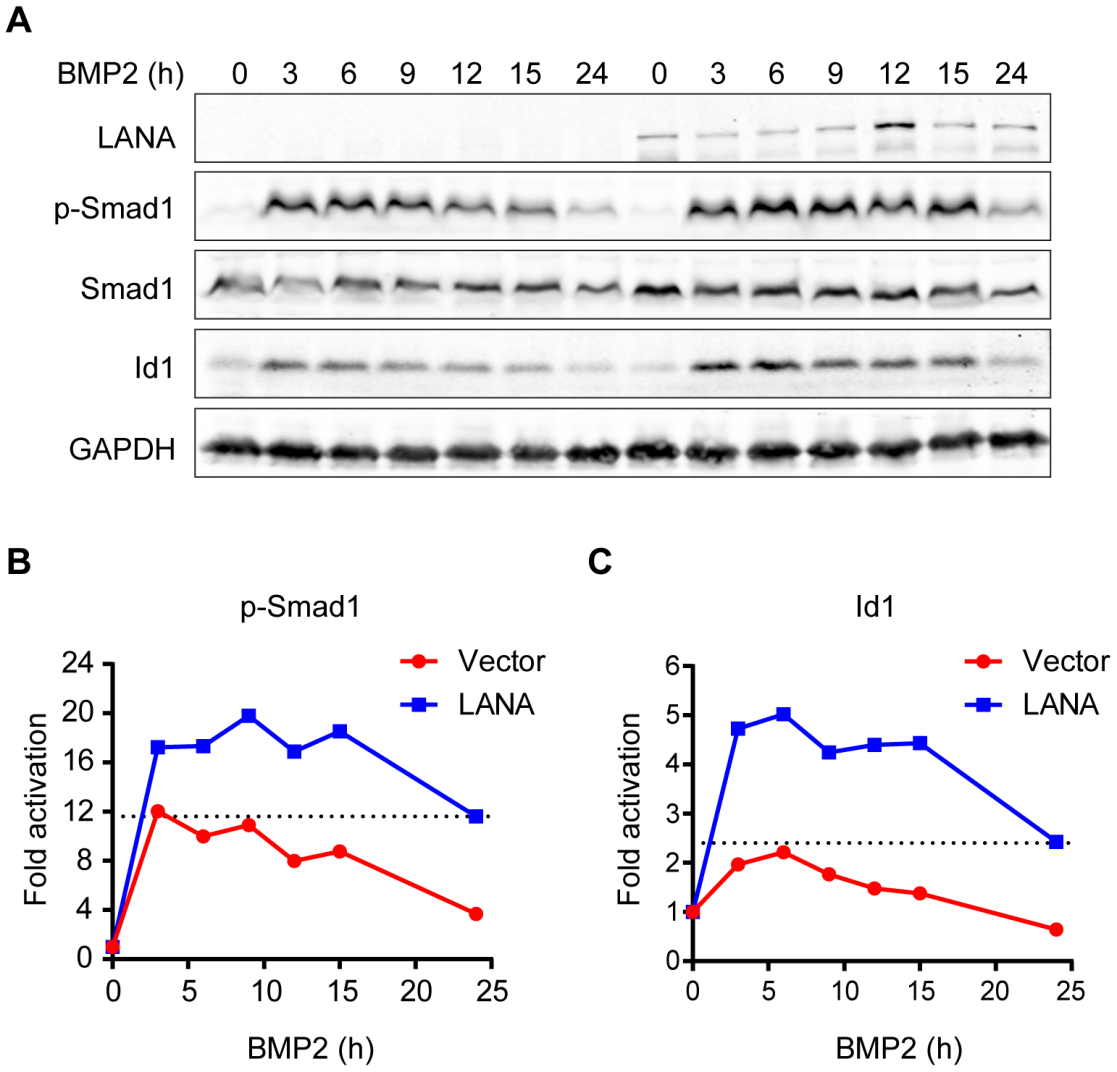
LANA sustained BMP-induced p-Smad1 and up-regulated Id1 expression. (A) LANA sustained BMP-induced p-Smad1 and up-regulated Id1 expression. SF-LANA or vector (2 µg each) transfected 293T cells in 35 mm dish were treated with BMP2 in a time course. Cells were harvested at indicated time and subjected to immunoblotting. Representative blots were shown in this panel. (B) The fold of activation of p-Smad1 at each time point was measured by calibrating the relative expression of p-Smad1 to the corresponding Smad1 and GAPDH expression, and then normalized to 0 hour. (C) The fold of activation of Id1 at each time point was measured by calibrating the relative expression of Id1 to the corresponding GAPDH expression, and then normalized to 0 hour.

BMP signaling regulates fundamental biological processes during embryonic development, postnatal development, as well as tumorigenesis [22]. The Smad1 MH2 domain is responsible for sensing BMP signaling, oligomer formation with other Smads, interaction with various DNA-binding proteins, and transcriptional activation of BMP downstream targets [18]. We wondered whether LANA modulated BMP-Smad1 signaling by regulating the expression and/or function of p-Smad1via interaction with Smad1-MH2 domain. To address this hypothesis, 293T cells were transiently transfected with LANA or a control vector and then treated with BMP2. 293T cells were harvested at different times and subjected to immunoblotting for the levels of p-Smad1 and BMP downstream target Id1. The levels of p-Smad1 activation and Id1 were normalized to their expression levels at 0 hour in two groups, respectively. Activation of p-Smad1 started to decline at 3 hours post BMP2 treatment and reached the basal level at 24 hours in the vector-transfected 293T cell while activation of p-Smad1 did not start to decline until 15 hours and continued to maintain at a relatively higher level at 24 hours in the LANA-transfected 293T cells (Fig. 2A, B). Therefore, BMP-induced p-Smad1 expression was significantly sustained by LANA (Fig. 2A, B). Consistent with these results, the induction of the canonical BMP downstream target Id1 was significantly potentiated in the LANA-transfected cells than the vector-transfected control cells by BMP2 (Fig. 2A, C).

Id1 was once reported to be up-regulated in KSHV-infected endothelial cells and in KS tissues; moreover, expression of LANA and vCyclin seemed to up-regulate Id1 expression in post-transcription level [16]. Since Id1 was well-recognized for its roles in tumorigenesis [13], [23], we sought to determine whether LANA up-regulated Id1 expression through the BMP-Smad1 pathway.

**Figure 3:**
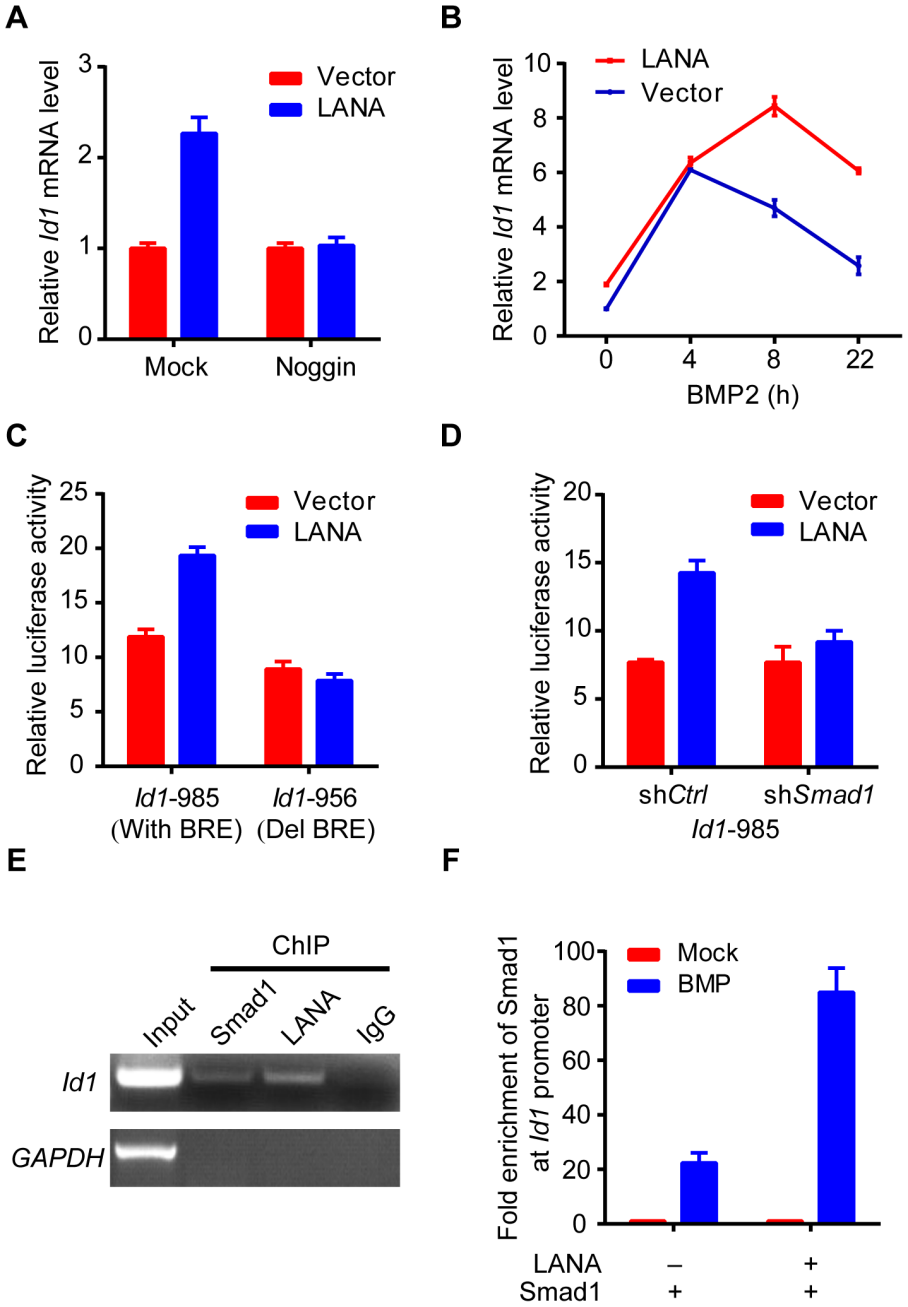
LANA up-regulated Id1 transcription in a BMP-Smad1 dependent manner. (A) Noggin inhibited Id1 up-regulation in LANA transfected 293T cells. LANA or vector transfected cells (2 µg each) were treated with noggin for 6 hours before harvest. Data were shown as mean ± s.e.m., n = 3. (B) BMP2 further enhanced LANA induction of *Id1* expression. LANA or vector transfected cells (2 µg each) were treated with BMP2 for indicated times before harvest. Data were shown as mean ± s.e.m., n = 3. (C) LANA up-regulated activity of *Id1*-985 but not *Id1*-956. A Smad1 binding site was contained in *Id1*-985 promoter region, but not in *Id1*-956. Data were shown as mean ± s.e.m., n = 3. (D) LANA did not up-regulate *Id1*-985 activity in 293T-sh*Smad1* cells. Data were shown as mean ± s.e.m., n = 3. (E) LANA was recruited to *Id1* promoter. SF-LANA and HA-Smad1 were co-transfected into 293T cells (12 µg each). Cells were treated with BMP2 for 2 hours before ChIP assay using indicating antibodies. (F) LANA significantly enhanced the enrichment of Smad1 at *Id1* promoter. HA-Smad1 was co-transfected with SF-LANA or vector (12 µg each). Cells were treated with BMP2 for 2 hours before ChIP assay using HA antibody.

As previously reported, we showed that Id1 was up-regulated in KSHV-infected human primary endothelial cells (Fig. S2). However, LANA but no other KSHV latent genes significantly up-regulated Id1 expression in 293T cells (Fig. S3). Meanwhile, LANA did not obviously alter Id1 protein stability. These results indicated that LANA regulated Id1 expression mainly at transcription level (Fig. S3). Consistent with these results, *Id1* transcription was up-regulated more than two fold in LANA-transfected 293T cells (Fig. 3A). Treatment with noggin, which inhibited BMP signaling, abolished LANA induction of *Id1* expression (Fig. 3A); while treatment with BMP2 further enhanced LANA induction of *Id1* expression (Fig. 3B). We then asked whether LANA was directly involved in *Id1* transcription regulation. In a promoter reporter assay, LANA increased the activity of *Id1* promoter reporter Id1-985, which contained a Smad1 binding site or BRE (BMP-responding element), but not that of the mutant reporter Id1-956 lacking the BRE [24] (Fig. 3C). Knock-down of Smad1 abolished LANA activation of the Id1-985 promoter reporter (Fig. 3D, Fig. S3). Therefore, LANA up-regulation of *Id1* transcription depended on the BMP-Smad1 signaling pathway.

We showed that LANA was directly recruited to the *Id1* promoter, together with Smad1 in ChIP-PCR assay (Fig. 3E). Moreover, LANA significantly enhanced the enrichment of Smad1 binding to the Id1 promoter after BMP2 treatment (Fig. 3F). Collectively, these results indicated that LANA promoted Smad1-mediated *Id1* transcription activation through sustaining p-Smad1 expression, and probably facilitating and extending the loading of Smad1 on *Id1* promoter.

Interestingly, we found that other Id family members, including Id2 and Id3 were also up-regulated in LANA-transfected 293T cells at both mRNA and protein levels (Fig. S4), whereas Id4 was not detected in our system. Furthermore, we showed that *Id2* and *Id3* were up-regulated in KSHV infected human primary endothelial cells (HUVECs) as *Id1* (Fig. S5). Knockdown of *Smad1* significantly impaired the expression of *Id1*, *Id2* and *Id3* in KSHV infected HUVECs, which suggested that Ids were mainly regulated by BMP-Smad1 pathway in those cells. We also showed that BMP signaling inhibitor Dorsomorphin dramatically repressed *Id1*, *Id2* and *Id3* in iSLK.219 cells (Fig. S5). Based on our data, we believed that LANA might generally up-regulated the transcription of Id family members through BMP-Smad1-Id signaling pathway in KSHV infected cells.

### Ids were aberrantly expressed in KS tissues and in KSHV-transformed cells

**Figure 4:**
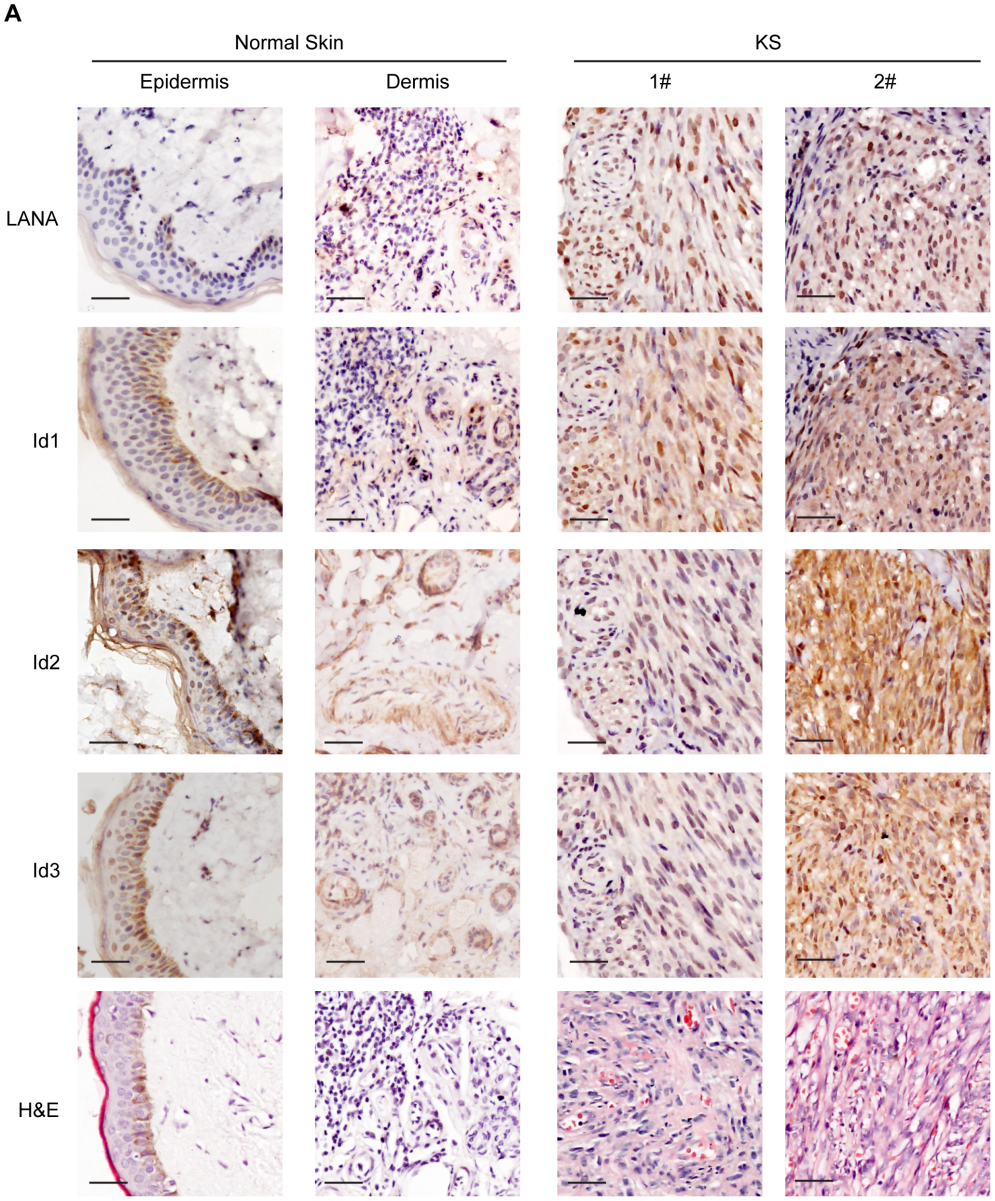
Ids were aberrantly expressed in KS tissues. Expression of Id1, Id2, Id3, and LANA were detected in 10 KS tissues and 5 normal skin tissues by immunohistochemistry. There were weak to modest staining signals of Id1, Id2 and Id3 only in the basal cells of epidermis and around the hair follicle of dermis in normal skin tissues. There was no LANA staining in any normal skin tissues. In contrast, there were strong staining signals of Id1, Id2, Id3 and LANA in the spindle tumor cells in KS lesions. Representative images of the immunohistochemistry staining were shown.

Since Ids were important oncogenic proteins, we sought to determine whether Ids were aberrantly expressed in KS tissues. We examined the expression of Id proteins and LANA in 10 cases of classical KS tissues and 5 cases of normal skin tissues by immunohistochemistry. As shown, there were weak to modest staining signals of Id1, Id2 and Id3 only in the basal cells of epidermis and around the hair follicle of dermis in normal skin tissues (Fig. 4). There was no LANA staining in any normal skin tissues (Fig. 4). In sharp contrast to normal skin tissues, there were strong staining signals of Id1, Id2 and Id3 in the spindle cells in the KS lesions. By staining for Ids and LANA in consecutive sections, positive signals of Ids were only observed in the spindle cells in KS lesions, which were also positive for LANA staining. No staining of Ids was observed in the adjacent tissues, which were negative for LANA (Fig. S6). Because of the small sample size, we were not able to perform a valid correlation analysis between Ids expression and the stage of the tumors. Nevertheless, our data suggested that Ids were aberrantly regulated in KS tumors and might be relevant to the development of KS. Because the p-Smad1 antibody was not suitable for immunohistochemical staining, we were not able to examine the expression of p-Smad1 in these KS lesions. Nevertheless, we showed that there was strong staining of Smad1 in the KS lesions but not in adjacent tissues (Fig. S6) indicating that BMP-Smad1-Id signaling might be involved in the aberrant expression of Ids in KS.

**Figure 5:**
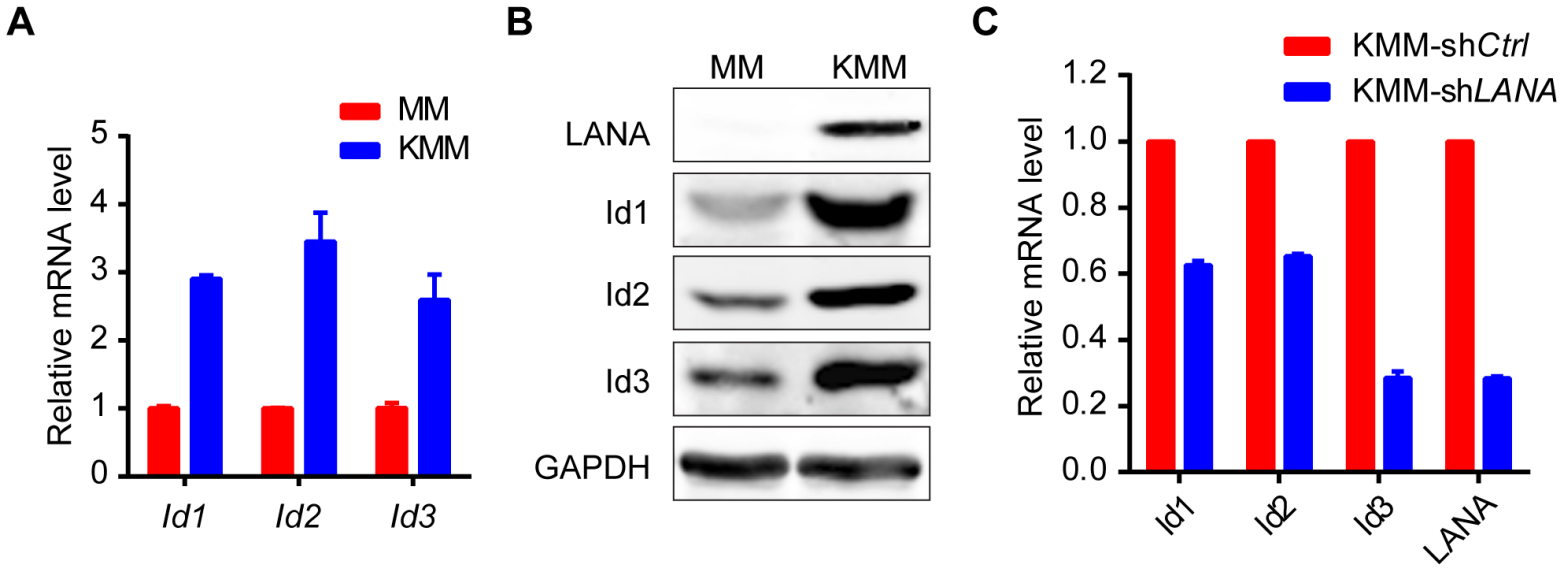
Ids were up-regulated in KSHV-transformed KMM cells. (A, B) Ids were up-regulated in KSHV-transformed MM cells (KMM) as shown by qRT-PCR (A) and immunoblotting (B). Data were shown as mean ± s.e.m., n = 3. (C) Knockdown of *LANA* in KMM cells decreased *Ids* expression. *Ids* and *LANA* expression was shown by qRT-PCR. Data were shown as mean ± s.e.m., n = 3.

KSHV can efficiently infect and transform primary rat embryonic metanephric mesenchymal precursor (MM) cells [7]. KSHV-transformed MM cells (KMM) efficiently induce tumors with virological and pathological features of KS [7]. We asked whether Id family members were up-regulated by LANA in KMM cells. We detected significantly higher levels of Id1∼Id3 (about 3 fold) in KMM cells than in MM cells at both mRNA and protein levels (Fig. 5A, B). Knock-down of LANA dramatically suppressed the expression of *Id1*, *Id2*, and *Id3* in KMM cells (Fig. 5C). These results indicated that LANA was responsible for the up-regulation of Ids in KMM cells.

### Ids were indispensable driving forces for KSHV-induced tumorigenesis

**Figure 6:**
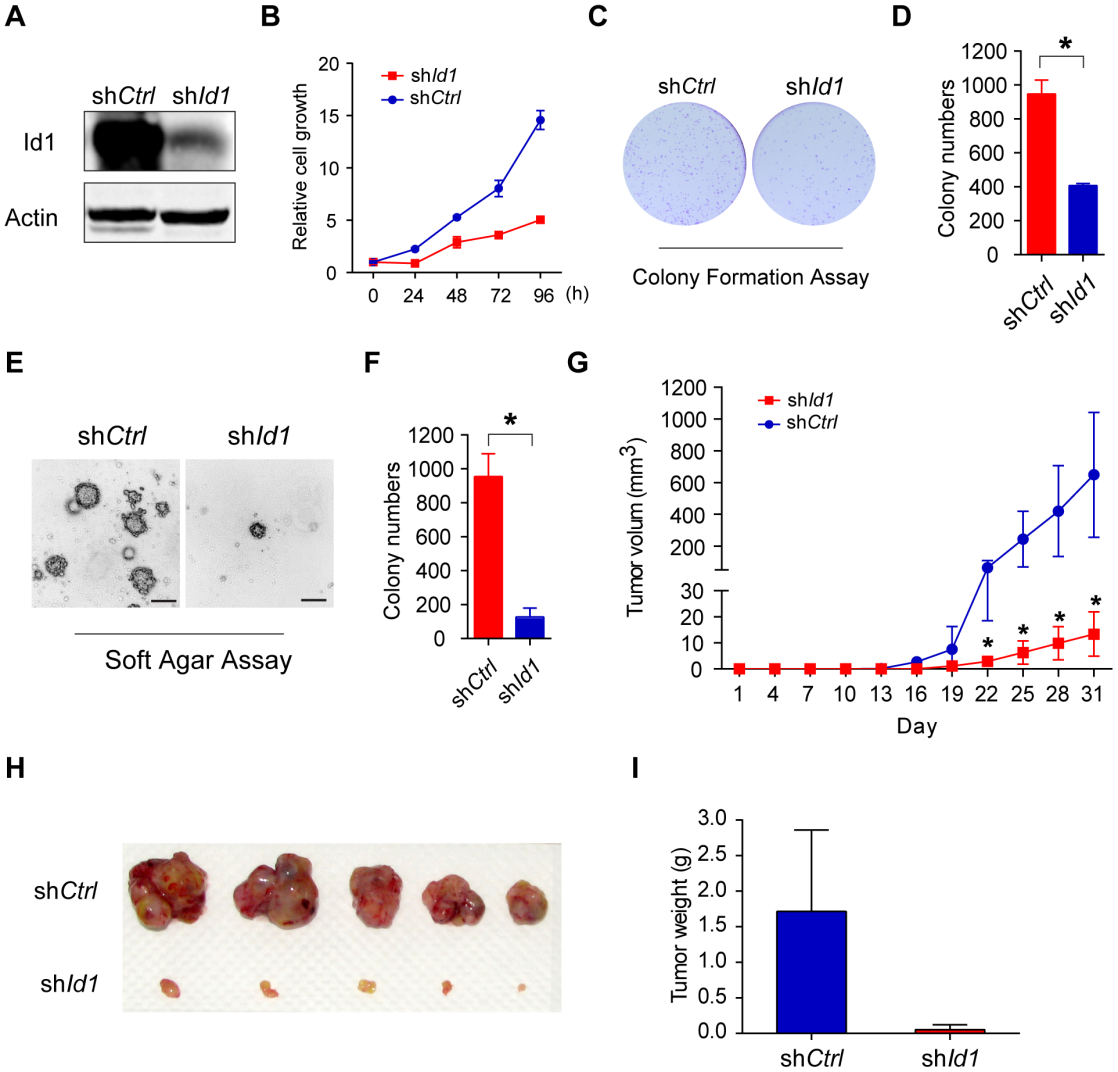
Knockdown of Id1 dramatically inhibited the tumorigenicity of KMM cells. (A) Expression of Id1 in KMM-sh*Ctrl* and KMM-sh*Id1* cells were checked by immunostaining. (B) KMM-sh*Id1* cells proliferate slower than KMM-sh*Ctrl* cells. Cell proliferation was measured by MTT assay. Data were shown as mean ± s.e.m., n = 3. (C, D) Knockdown of *Id1* inhibited the colony formation ability of KMM cells. Cells were stained by crystal violet. Colony numbers were counted by Quantity One software. Data were shown as mean ± s.e.m., n = 3. * p<0.05. (E, F) Knockdown of *Id1* inhibited anchorage-independent growth of KMM cells. Cells were stained by crystal violet. Colony numbers were counted by Quantity One software. Data were shown as mean ± s.e.m., n = 3. * p<0.05. (G, H, I) Knockdown of *Id1* inhibited tumor growth of KMM cells *in vivo*. 1×10^6^ KMM-sh*Ctrl* cells or KMM-sh*Id1* cells were subcutaneously injected into nude mice. Tumor volume was measured every 3 days. Tumor volume was calculated by the formula: (length×width^2^)/2. Data were shown as mean ± s.e.m., n = 5. * p<0.05.

Id proteins, especially Id1, are able to promote cell proliferation and cell cycle progression. To determine if LANA deregulation of the BMP-Smad1-Id pathway could contribute to KSHV-mediated tumorigenesis [7], we established KMM-sh*Id1* cell lines with high *Id1* knockdown efficiency and determined the effect on cellular transformation (Fig. 6A). Knock-down of *Id1*dramatically decreased the proliferation of KMM cells (Fig. 6B) and inhibited the formation of foci in culture (Fig. 6C, D), formation of colonies in soft agar (Fig. 6E, F), and induction of tumors in nude mice (Fig. 6G, H, I) In contrast, knockdown of *Id1* in MM cells only slightly decreased the proliferation of MM cells (Fig. S7). We also established KMM-sh*Id*2 and KMM-sh*Id*3 cell lines (Fig. S8). Knockdown of *Id2* and *Id3* inhibited anchorage-independent growth of KMM cells in soft agar (Fig. S8). Moreover, knockdown of either LANA or Smad1 also severely impaired the anchorage-independent growth of KMM cells (Fig. S9). These results indicated that Ids were required for maintaining the oncogenic phenotype of KMM cells.

We further asked whether Ids were the driving force for KSHV-mediated cellular transformation. We overexpressed Id1 in MM cells, however, no direct cellular transformation was observed as expected (Fig. S10). Nevertheless, ectopic expression of Id1 in KMM cells (Fig. S11A) further accelerated cell proliferation (Fig. S11B), and increased the formation of foci in culture and formation of colonies in soft agar (Fig. S11C, D, E, F).

Collectively, our data provided evidence that LANA increased BMP-Smad1-Id signaling and this pathway was required for KSHV-induced tumorigenesis.

### Targeting BMP-Smad1-Id pathway inhibited the growth of KSHV-induced tumors

**Figure 7:**
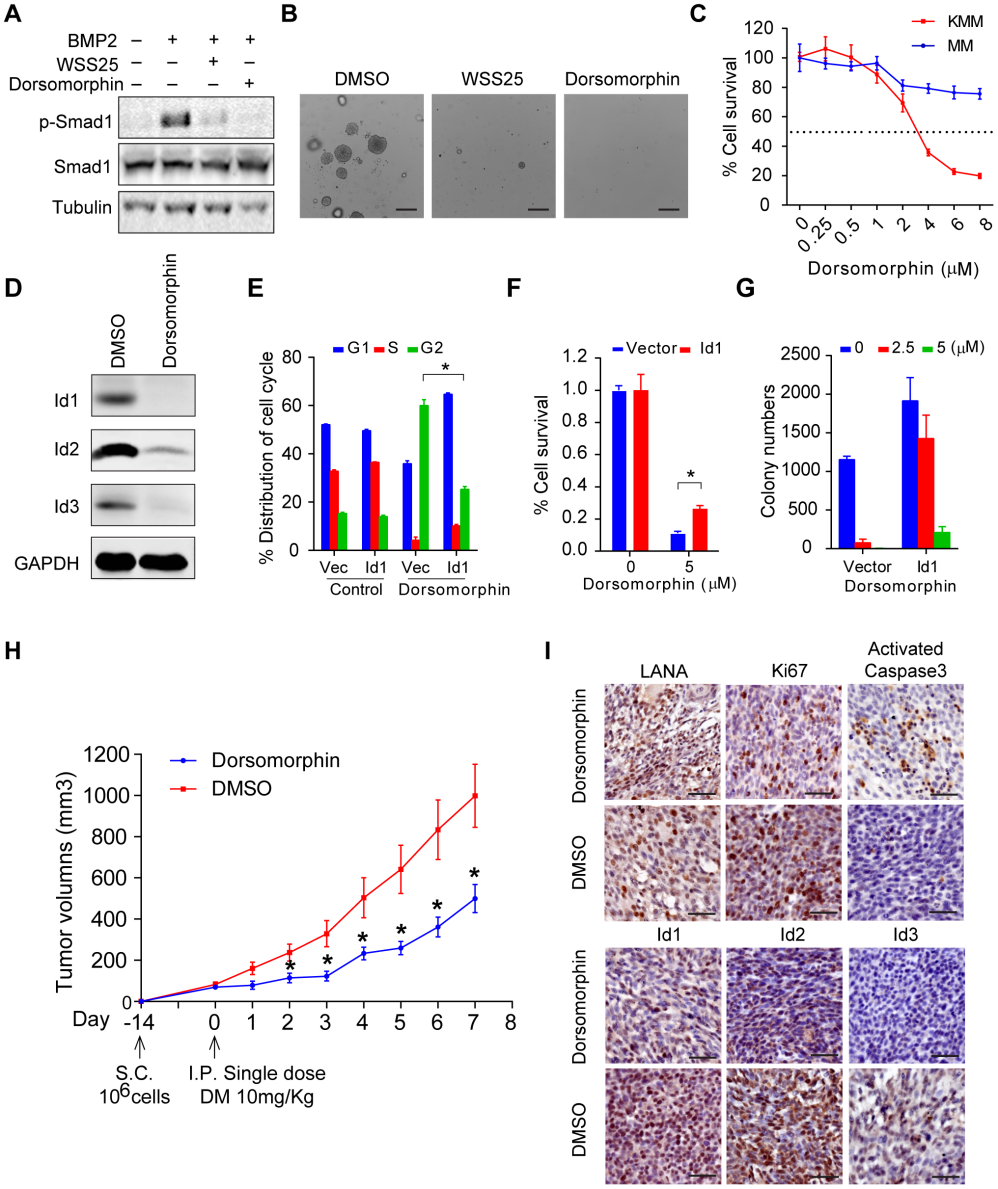
Dorsomorphin inhibited tumorigenesis of KMM cells mainly through targeting BMP-Smad1-Id pathway. (A) WSS25 and Dorsomorphin dramatically inhibited BMP2-stimulated p-Smad1 expression. KMM cells were treated with BMP2 (10 ng/ml), with or without Dorsomorphin (5 µM), WSS25 (40 mg/ml) as indicated for 2 hours before harvest for immunoblotting. (B) WSS25 and Dorsomorphin inhibited the anchorage-independent cell growth of KMM cells. Dorsomorphin (5 µM), or WSS25 (40 mg/ml) was supplemented during soft agar assay. Colony numbers were counted by Quantity One software. Data were shown as mean ± s.e.m., n = 3. * p<0.05. (C) Dorsomorphin showed preferential toxicity to KMM cells. KMM cells or MM cells were seeded 4000 cells/well. 24 hours after seeding, medium was replaced with Dorsomorphin medium as indicated. Cell viability was measured by MTT assay 48 hours post Dorsomorphin treatment. Data were shown as mean ± s.e.m., n = 3. (D) Dorsomorphin dramatically inhibited the expression of Ids. KMM cells were treated with 5 µM Dorsomorphin for 24 hours before harvesting. Expressions of Ids were detected by immunoblotting. (E) Ectopic expression of Id1 significantly rescued Dorsomorphin induced G2/M arrest. KMM-Vector and KMM- Id1 cells were treated with DMSO or 5 µM Dorsomorphin for 48 hours. Then the cells were harvested and subjected to PI staining and cell cycle analysis by Mod Fit software. Data were shown as mean ± s.e.m., n = 3. * p<0.05. (F) Ectopic expression of Id1 significantly rescued Dorsomorphin induced cellular toxicity in KMM cells. KMM-Vector and KMM-Id1 cells were seeded and treated with Dorsomorphin (5 µM) as (C). Data were shown as mean ± s.e.m., n = 3. * p<0.05. (G) Ectopic expression of Id1 rescued Dorsomorphin-inhibited anchorage-independent growth of KMM cells. Colony numbers were counted by Quantity One software. Data were shown as mean ± s.e.m., n = 3. (H) Dorsomorphin significantly inhibited the tumor growth of KMM cells *in vivo*. 1×10^6^ KMM cells were subcutaneously injected into nude mice. When tumor volume reached about 50∼100 cm^3^, nude mice were divided into two groups randomly. One group was introperitoneally injected with a single dose of Dorsomorphin (10 mg/Kg), the other group was injected with vehicle. Tumor volume was monitored daily and calculated by the formula: (length×width^2^)/2. Data were shown as mean ± s.e.m., n = 5. * p<0.05. (I) Representative Immunohistochemical staining images of LANA, Ki67, activated caspase 3, Id1, Id2 and Id3 in xenograft tumors were shown.

Based on the above findings, we speculated that inhibitors of the BMP pathway might be potential therapeutic agents of KS. Dorsomorphin potently inhibits BMP-mediated Smad1/5/8 phosphorylation [25], while WSS25 disrupts the interaction between BMP and BMP receptor [26], [27]. Indeed, treatment with these two molecules dramatically inhibited BMP2-stimulated p-Smad1 expression (Fig. 7A), and inhibited the anchorage-independent cell growth of KMM cells in soft agar assay (Fig. 7B). Importantly, compared to MM cells, Dorsomorphin showed preferential toxicity to KMM cells (Fig. 7C), indicating Dorsomorphin selectively targeted KSHV-transformed cells. Furthermore, we found that Dorsomorphin dramatically inhibited the expression of Ids (Fig. 7D) while ectopic expression of Id1 significantly rescued Dorsomorphin induced G2/M arrest [28], cellular toxicity in KMM cells (Fig. 7E, F and Fig. S12), and partially rescued Dorsomorphin inhibition of anchorage-independent colony formation (Fig. 7G). These results indicated that Dorsomorphin mainly inhibited the oncogenicity of KMM cells through targeting the BMP-Smad1-Id pathway. To strengthen our conclusion, we showed that overexpression of Id1 significantly rescued Dorsomorphin-induced cellular toxicity in 293T cells in a dose-dependent manner (Fig. S13).

Finally, we determined the efficacy of Dorsomorphin in inhibiting *in vivo* tumor growth of KMM cells. We subcutaneously injected 1×10^6^ KMM cells into BALB/c nude mice. When tumor volume reached about 50∼100 cm^3^, the nude mice were randomly divided into 2 two groups. One group was intraperitoneally injected with a single dose of Dorsomorphin at 10 mg/Kg [29] while the other group was injected with vehicle control. Impressively, single treatment with Dorsomorphin was sufficient to significantly inhibit the tumor growth of KMM cells (Fig. 7H). Immunohistochemical staining showed that Dorsomorphin inhibited Id1, Id2, Id3 and Ki67 expression and activated caspase 3 in the tumors (Fig. 7I). Interestingly, LANA remained positive in the Dorsomorphin-treated tumors (Fig. 7I), suggesting that the antitumor activity of Dorsomorphin was not dependent on the inhibition of KSHV infection and replication in KMM cells.

## Discussion

**Figure 8:**
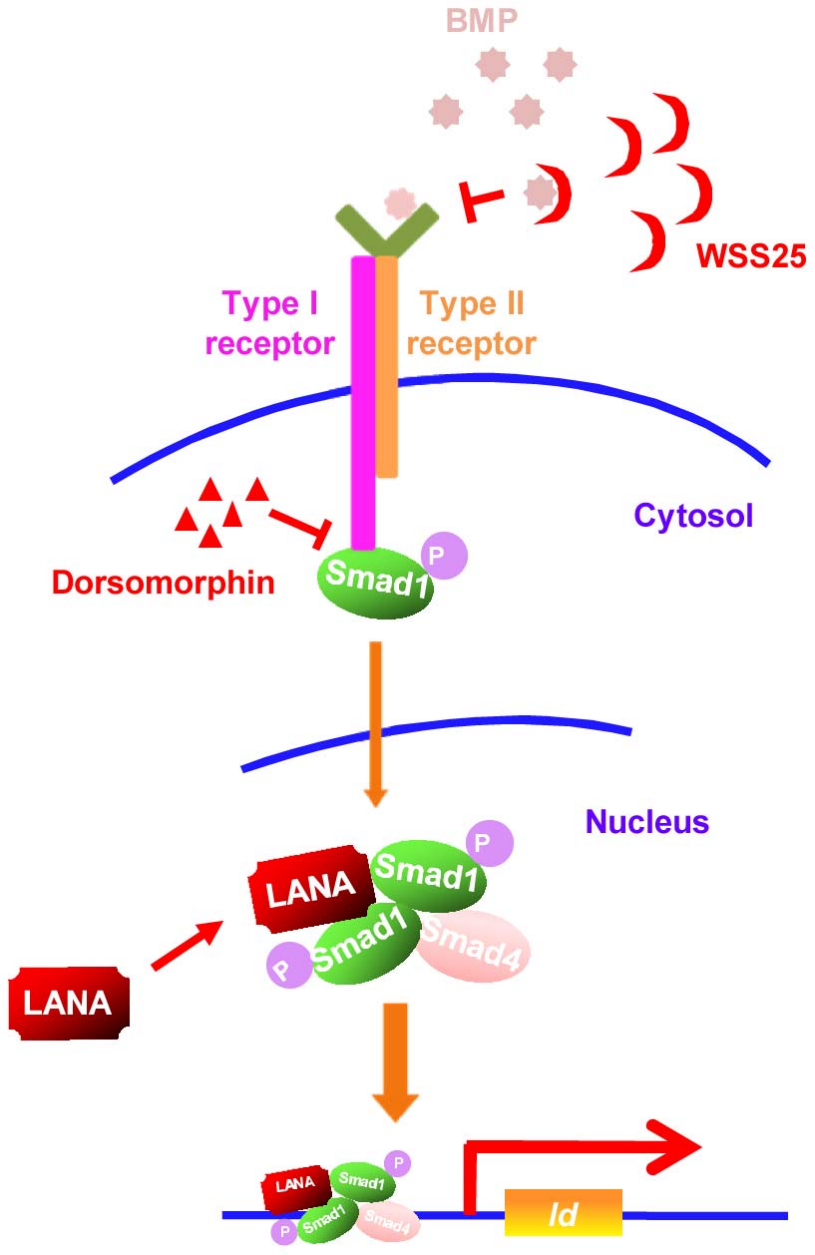
A schematic working model. Small inhibitors targeting BMP-Smad1-Id signaling pathway through different mechanisms may serve as potential therapeutics of KS by targeting Id expression.

Our results showed that KSHV LANA interacted with BMP-activated p-Smad1 in the nucleus, sustained p-Smad1 expression, and facilitated its loading on the *Id* promoter leading to aberrant expression of Ids, which were indispensable driving forces for KSHV-induced tumorigenesis. Thus, KSHV hijacks and converts a developmental pathway into an oncogenic pathway, which is essential for KSHV-induced transformation. Furthermore, our results have shown that small inhibitors targeting BMP-Smad1-Id signaling pathway may serve as potential candidates for the treatment of KS (summary in Fig. 8).

Although KSHV exerts multiple mechanisms to promote cell survival by repressing TGF-β signaling [30]–[32], little is known whether KSHV manipulates BMP signaling and contribute to the pathogenesis of KSHV-induced malignancies. Previously, KSHV lytic gene K5 was reported to inhibit BMP signaling by down-regulating BMPR-II through ubiquitination-mediated degradation [33]. However, KSHV is predominantly maintained in the latent state of replication in KS spindle tumor cells and KMM cells, in which K5 is usually not expressed. In this context, we believe that KSHV hijacks the BMP-Smad1-Id pathway to promote tumorigenesis.

We previously reported that Smad1 was not detected in PEL cells [31] and LANA did not interact with Smad5. It is unlikely that LANA is involved in Id regulation in PEL cells. We showed that Id1∼3 were expressed in KSHV-positive BCBL1 and BC3 cells at levels similar to KSHV-negative BJAB cells (Fig. S14). Since BJAB was not an ideal control for BCBL1 and BC3 cells, we further compared the expression of Ids in BJAB and KSHV stably transfected BJAB (KSHV-BJAB) cells. We found that *Id2* and *Id3*, but not *Id1* was decreased by about 50% in KSHV-BJAB cells compared to BJAB cells (Fig. S14). Since Id2 and Id3 but not Id1 were reported to be down-regulated in the vFLIP-transfected cells [34], vFLIP might be the main viral gene that regulates the expression of Ids in PEL cells.

Since Id1∼3 are significantly up-regulated in KS lesions compared to adjacent tissue and normal skin and in KMM cells compared to MM cells, up-regulation of Ids by LANA through LANA-Smad1-Id signaling is likely the principal mechanism that KSHV regulates the expression of Ids in KS tumor cells and in KSHV-transformed KMM cells. Interestingly, Id1 and Id3 were induced by EBV latent protein LMP1 [35], [36]. LMP1 inactivates the function of Foxo3a leading to up-regulation of Id1. Id1 increased cell proliferation and conferred resistance to TGFβ-mediated cell cycle arrest in nasopharyngeal epithelial cells [37]. Therefore, Id proteins may serve as conserved targets for oncogenic herpesviruses.

Ids inhibit apoptosis and promote cell proliferation through distinct mechanisms [13]. For example, Id1 had been shown to inhibit E-protein and Ets-protein-mediated activation of the p16/INK4a [38], [39]. Id2 has been found to reverse cellular growth inhibition by the retinoblastoma protein (pRb) through direct interaction with pRb [40]. How individual Ids promote KSHV-mediated oncogenesis remain to be further clarified. Our data showed that LANA up-regulated BMP-Smad1-Id signaling was required but not sufficient for KSHV-induced tumorigenesis. It is likely that additional oncogenic signaling pathways are involved in KSHV-induced cellular transformation and tumorigenesis. Discovery of these additional pathways could help better understanding of how KSHV induces tumorigenesis.

Dorsomorphin is known to potently inhibit the expression of Ids through suppressing BMP-induced Smad1 phosphorylation. Our results showed that Dorsomorphin dramatically inhibited the growth of KMM cells *in vitro* and tumor growth *in vivo*. Even though Dorsomorphin might also target other kinases [41], our results showed that Id1 was capable of rescuing Dorsomorphin-induced G2/M arrest and cellular toxicity in KMM cells. Therefore, we have demonstrated that, by targeting the KSHV-deregulated BMP-Smad1-Id pathway, Dorsomorphin inhibits KSHV-induced tumorigenesis. Dorsomorphin might be a promising lead compound for KS therapy.

Currently, it is still unknown how LANA sustains p-Smad1 activation through their interaction. In the basal state, Smad1 constantly shuttles between cytoplasm and nucleus through its N-terminal nuclear localization signal (NLS) motif and C-terminal nuclear export signal (NES) [42]. Upon activation by BMP, the C-terminal of Smad1, which is phosphorylated at SXS motif, undergoes conformation change, and creates an acidic knob to form a trimer with the homologous MH2 domain of another Smad1 molecule and MH2 domain of Smad4 [43]. The ligand-induced phosphorylation promotes the accumulation of the hetero-oligomer in the nucleus by inhibiting the nuclear export and enhancing its import [42]. In the nucleus, Smad1/Smad4 complexes bind to other co-transcription factors and initiate target gene transcription. The signal undergoes rapid termination through dephosphorylation in its C-terminal SXS motif by PPM1A [44] and/or SCP (small C-terminal domain phosphatase) family of nuclear phosphatases [45] or degradation via polyubiquitylation and proteasome-mediated degradation by Smurf1/2 [46] or CHIP [47]. In this study, we have demonstrated the interaction between the N-terminal of LANA and MH2 domain of Smad1. This interaction may have several effects on sustaining the activated Smad1 in the nucleus: 1) it may stabilize the heteromeric complex between the phosphorylated Smad1 and the common mediator Smad4, thus masking the NES motif; 2) it may protect Smad1 from dephosphorylation caused by PPM1A or SCPs; 3) it may disrupt the rapid Smad1 turnover via Ubiquitin-Proteasome Pathway mediated by Smurf1/2 or CHIP. Thus, LANA facilitates the loading of functional p-Smad1 on the Id promoter and ultimately leads to aberrant expression of Ids. However, additional works are required to confirm any of these speculations.

In a summary, our study has revealed that BMP-Smad1-Id signaling pathway is positively regulated by LANA and serves as an intrinsic oncogenic pathway of KSHV-induced tumorigenesis. More importantly, we have shown that the BMP-Smad1-Id pathway is a potential therapeutic target for KS.

## Materials and Methods

### Ethics statement

The clinical section of the research was reviewed and ethically approved by the Institutional Ethics Committee of the First Teaching Hospital of Xinjiang Medical University (Urumqi, 127 Xinjiang, China; Study protocol # 20082012). Written informed consent was obtained from all participants, and all samples were anonymized. All participants were adults.

The animal experiments were approved by the Institutional Animal Care and Use Committee of the Institut Pasteur of Shanghai, Chinese Academy of Sciences (Animal protocol # A2013010). All animal care and use protocols were performed in accordance with the Regulations for the Administration of Affairs Concerning Experimental Animals approved by the State Council of People's Republic of China.

### Cell lines, plasmids, antibodies and reagents

Rat embryonic metanephric mesenchymal precursor cells (MM cells), KSHV-transformed MM cells (KMM), 293T cells were maintained in DMEM (HyClone) supplemented with 10% fetal bovine serum (HyClone). HUVEC was maintained in EGM (Lonza). KMM/sh*smad1*, KMM/sh*Id1*, KMM/sh*LANA*, and KMM/sh*Control*, KMM/Id1, KMM/Vector, 293T/sh*Smad1*, 293T/sh*Control*, 293T/SF-LANA and 293T/SF-Puro cell lines were established by infection of indicated lentivirus according to the manufacturer's instructions (System Bioscience).

LANA truncation plasmids were previously reported [48]. pCDH-SF-*LANA* was constructed by sub-cloned full-length *LANA* into pCDH-SF-EF1-*Puro* by EcoRI and BamHI sites. Reporter plasmids pGL3-*Id1*-985 and pGL3-*Id1*-956 were constructed as previously reported [24]. HA-Smad1and Flag-Smad1, were provided by Dr. Naihe Jing (Shanghai Institutions of Biological Sciences). Sh*LANA* was previously reported [49]. sh*Id1*, sh*Id2*, sh*Id3* and sh*Smad1* were constructed in pLKO.1 using the following targeting sequence: *Id1* (AAGGTCACATTTCGTGCTTCT); *Id2* (CAGCACGTCATCGATTATATC); *Id3* (GTGATCTCCAAGGACAAGAGG) ; *Smad1*(CGGTTGCTTATGAGGAACCAA). Truncated or SXS motif mutated *Smad1* plasmids were constructed by cloning the indicated sequence into pCMV-HA vector using the following primers:

Smad1-N (F: 5′-CGCGTCGACAATGAATGTGACAAGTTTATT-3′, R: 5′-CGCCTCGAGTTAAGCAACCGCCTGAACATCTC-3′);

Smad1-C (F: 5′-CGCGTCGACAATGCCTGTACTTCCTCCTGTGCT-3′, R: 5′-CGCCTCGAGTTAAGATACAGATGAAATAGGAT-3′);

Smad1-MH2 (F: 5′-CGCGTCGACAATGTATGAGGAACCAAAACACTG-3′, R: 5′-CGCCTCGAGTTAAGATACAGATGAAATAGGAT-3′);

Smad1-DVD (F: 5′-CGCGTCGACAATGAATGTGACAAGTTTATT-3′, R: 5′-CGCCTCGAGTTAATCTACATCTGAAATAGGATTA-3′);

Smad1-AVA (F: 5′-CGCGTCGACAATGAATGTGACAAGTTTATT-3′, R: 5′-CGCCTCGAGTTAAGCTACAGCTGAAATAGGATT-3′);

Smad1-ΔC3 (F: 5′-CGCGTCGACAATGAATGTGACAAGTTTATT-3′, R: 5′-CGCCTCGAGTTAAGCTACAGCTGAAATAGGATT-3′).

Expression plasmids of truncated Smad1-MH2 were constructed by cloning the indicated sequence into pEGFP vector using the following primers:

MH2-N (F: 5′-cgcAGATCTTATGAGGAACCAAAACACTG-3′, R: 5′-cgcGGATCCTTAATGATGGTAGTTGCAGTTCC-3′)

MH2-M (F: 5′-cgcAGATCTCGTTTCTGCCTTGGGCTGCT-3′, R: 5′-cgcGGATCCTTATGTAAGCTCATAGACTGTCTCA-3′)

MH2-C (F: 5′-cgcAGATCTGGATTTCATCCTACTACTGTTTGC-3′, R: 5′-cgcGGATCCTTAAGATACAGATGAAATAGG-3′)

MH2-F (F: 5′-cgcAGATCTTATGAGGAACCAAAACACTG-3′, R: 5′-cgcGGATCCTTAAGATACAGATGAAATAGG-3′).

The antibodies and reagents were used as follows: anti-LANA (1B5, prepared in our lab), anti-Smad1 (Santa cruz, sc-7965x), anti-pSmad1/5/8 (Cell signaling technology, #9511), anti-Id1 (Santa cruz, sc-488), anti-Id2 (Santa cruz, sc-489), anti-Id3(Santa cruz, sc-490), anti-Ki67 (Novocastra, NCL-Ki67p), anti-cleaved Caspase-3 (Cell signaling technology, #9661). Anti-Flag M2 affinity gel (Sigma, A2220), Strep-Tactin sepharose (IBA, 2-1201-010), desthiobiotin (IBA, 2-1000-001), BMP2 (Sigma, B3555), Cycloheximide (Sigma, C1988), Dorsomorphin (Sigma, P5499) and WSS25 were kindly provided by Dr. Kan Ding from Shanghai Institute of Materia Medica [26].

### Tandem affinity purification (TAP)

TAP of SF-LANA was done as previously described [19]. Briefly, 293T-SF-LANA or 293T-SF-Puro cells were harvested and subjected to nuclear extraction as previously described [50]. Dialyzed nuclear extract was loaded into a column of prewashed Strep-Tactin Superflow (0.5 ml bed volume, IBA). The column was washed with 10 bed volume of Buffer W (50 mM Tris pH 7.9, 100 mM KCl, 10% Glycerol, 0.2 mM EDTA, 0.5 mM DTT, 0.1% Triton-X100, 0.2 mM PMSF) and eluted with 3 bed volume of Buffer E (Buffer W containing 2.5 mM D-desthiobiotin). The elute was then subjected to second round of affinity purification by anti-Flag M2 affinity gel for 2 hours at 4°C. The beads were washed with Buffer W for 5 times and eluted with 3×Flag Peptide in Buffer W. The elute was monitored by SDS-PAGE and subjected to mass spectrometry.

### Coimmunoprecipitation (co-IP) and immunoblotting

Cells were lysed in radio immunoprecipitation assay (RIPA) buffer (50 mM Tris [pH 7.6], 150 mM NaCl, 2 mM EDTA, 1% Nonidet P-40, 0.1 mM PMSF, 1×phosphatase inhibitors [Phospho-Stop, Roche]) for 1 h on ice with brief vortexing every 10 min. The lysate were incubated with antibody or affinity beads as indicated overnight at 4°C. The immunoprecipitations were separated by SDS-PAGE and analyzed by immunoblotting. For cytoplasmic protein and nuclear protein fractionation, cells were harvested and extracted as described [51].

### Real-time RT-PCR

Cells were collected and lysed in Trizol buffer (Life technology), and RNA was isolated according to the manufacturer's instructions. Reverse transcription was performed with a cDNA Reverse Transcription Kit (Toyobo). Real-time RT-PCR was performed with a SYBR green Master Mix kit (Toyobo). Relative mRNA levels were normalized to *Actin* and calculated by ΔΔCT method. The primers were listed below:

*Id1* (F: 5′-CTGCTCTACGACATGAACGG-3′, R: 5′-GAAGGTCCCTGATGTAGTCGAT-3′);

*Id2* (F: 5′-GCTATACAACATGAACGACTGCT-3′, R: 5′-AATAGTGGGATGCGAGTCCAG-3′);

*Id3* (F: 5′-GAGAGGCACTCAGCTTAGCC-3′, R: 5′-TCCTTTTGTCGTTGGAGATGAC-3′);

*Actin* (F: 5′-GCACGGCATCGTCACCAACT-3′, R: 5′-CATCTTCTCGCGGTTGGCCT-3′).

### Chromatin immunoprecipitation assay (ChIP)

Chromatin immunoprecipitation (ChIP) was performed as previously described. Briefly, 5 µg correspondent antibody (anti-HA mAb, anti-Flag-mAb) or control mouse immunoglobulin (IgG) was added into each group of lysate at 4°C overnight. Then 50 µl proteinA/G beads, which had been precleared with binding buffer containing 0.2 mg of salmon sperm DNA per ml for 6 h, were added into each sample at 4°C for 2 h for immunoprecipitation. To extract the DNA fragment, TE buffer with 1% SDS and proteinase K (Beyotime) was added to the washed precipitates. After incubation at 65°C for at least 6 h, the eluted solution was subjected to DNA extraction kit (Bio-Dev). Specific primers used for chromatin immunoprecipitation (ChIP) DNA amplification matched the Id1 promoter region were: *Id1*-F: 5′-CAGTTTGTCGTCTCCATG-3′; *Id1*-R: 5′-TCTGTGTCAGCGTCTGAA-3′; *GAPDH*-F: 5′-TACTAGCGGTTTTACGGGCG-3′; *GAPDH*-R: 5′-TCGAACAGGAGGAGCAGAGAGCGA-3′.

### Functional assay

MTT assay for cell proliferation or toxicity was conducted according to the manufacturer's instructions (Beyotime). For cell proliferation, 1000 cells were seeded per well in 96-well plates as indicated; for toxicity, 4000 cells were seeded per well in 96-well plates with DMEM containing Dorsomorphin of indicated concentrations.

Cell cycle assay was conducted according to manufacturer's instructions (Beyotime). KMM-Vector and KMM-Id1 cells were treated with DMSO or 5 mM Dorsomorphin for 48 hours. Then the cells were harvested and subjected to PI staining and cell cycle analysis by Mod Fit software.

Soft agar assay: Six-well plates were covered with a bottom layer of 1% agar (Invitrogen) in DMEM containing 10% FBS. Then 10000 cells were prepared in DMEM containing 10% FBS and 0.4% agar and seeded onto the solidified bottom layer. After two weeks of cell culture, colonies were photographed by microscopy and stained with 0.005% crystal violet. The number of colonies was analyzed by Quantity One.

Colony formation assay: 1000 cells were prepared in DMEM containing 10% FBS and seeded in six-well plates. After two weeks of cell culture, colonies were photographed by microscopy and stained with 0.005% crystal violet. The number of colonies was analyzed by Quantity One.

### Histopathology and immunohistochemistry (IHC) analysis

The clinical tissue specimens from 10 patients with KS were collected from Xinjiang province, northwestern of China. The clinical section of the research was reviewed and ethically approved by the Institutional Ethics Committee of the First Teaching Hospital of Xinjiang Medical University (Urumqi, 127 Xinjiang, China; Study protocol # 20082012). The expression of LANA, Id1, Id2, Id3, Smad1, Ki67, and activated caspase 3 were analyzed by IHC as described [52].

### Xenograft experiment

1×10^6^ KMM-sh*Ctrl* cells or KMM-sh*Id1* cells were subcutaneously injected into BALB/c Nude mice. There were 5 mice in each group. The size of tumor was measured every 3 day. Tumor volume was calculated by the formula: (length×width^2^)/2. Nude mice were sacrificed at the same time when the size of tumors in sh*Ctrl* group reaches about 2000 mm^3^. In another xenograft assay with drug treatment, 1×10^6^ KMM cells were subcutaneously injected into BALB/c Nude mice. When tumor volume reached about 50∼100 cm^3^, nude mice were divided into 2 two groups randomly. There were 5 mice in each group. One group was intraperitoneal injection with a single dose of Dorsomorphin (10 mg/Kg), the other group was injected with vehicle. Tumor volume was monitored daily and calculated by the formula: (length×width^2^)/2. These animal experiments were approved by the Institutional Animal Care and Use Committee of the Institut Pasteur of Shanghai, Chinese Academy of Sciences (Animal protocol # A2013010).

### Statistical analysis

Data were analyzed by Student's t test. P<0.05 was considered to be significant (two tailed). Error bars represent standard error of mean (s.e.m.).

### Accession numbers

Gene IDs:

BMP2: 650

Smad1: 4086

Smad5: 4090

Id1: 3397

Id2: 3398

Id3: 3399

LANA: 4961527

## Supporting Information

Figure S1 LANA interacted with Smad1 but not Smad5. (A) LANA interacted with Smad1 in KSHV infected cells. 293T-KSHV.219 cells were harvested for endogenous co-IP and immuoblotting as indicated. (B) N-terminal of LANA was responsible for Smad1 binding. HA-Smad1 (12 µg) was co-transfected with different truncated LANA constructs, Intracellular Notch or vector (12 µg each) into 293T cells. Cell lysates were immunoprecipitated as indicated. (C) LANA did not interact with Smad5. HA-Smad5 (12 µg) was co-transfected with SF-LANA or vector (12 µg each) into 293T cells. Cell lysates were immunoprecipitated as indicated.
(TIF)

Figure S2 Id1 was up-regulated in KSHV-infected human primary endothelial cells. (A) HUVECs were harvested for immunoblotting as indicated at 24 hours post KSHV infection. (B) HUVECs were harvested for qRT-PCR as indicated at 24 hours post KSHV infection.
(TIF)

Figure S3 LANA up-regulated *Id1* expression in transcription level. (A) LANA did not alter Id1 stability in 293T cells. LANA or vector (12 µg each) transfected 293T cells were treated with 5 µg/ml CHX. Cells were harvested at the indicated times. Cell lysates were analyzed by immunoblotting. (B) Relative expression of Id1 after CHX treatment was quantified. (C) LANA but no other latent genes were responsible for Id1 up-regulation. vFLIP, vCyclin, LANA, miR-Cluster or Vector (12 µg each) were transfected into 293T cells. Cell lysates were analyzed by immunoblotting. (D) Expression of Smad1 in 293T-sh*Smad1* and 293T-sh*Ctrl* cells was detected by immunoblotting.
(TIF)

Figure S4 Ids were up-regulated in LANA transfected 293T cells in both mRNA level (A) and protein level (B).
(TIF)

Figure S5 Ids were generally up-regulated in KSHV infected cells through BMP-Smad1 signaling pathway. (A) Expression of Ids was up-regulated in KSHV infected HUVECs. (B) Knockdown of Smad1 significantly impaired the expression of *Id1*, *Id2* and *Id3* in KSHV infected HUVECs. (C) Knockdown efficiency of si*Smad1* was checked by qRT-PCR. (D) Dorsomorphin dramatically repressed *Id1*, *Id2* and *Id3* in iSLK.219 cells.
(TIF)

Figure S6 Expression of Ids, LANA and Smad1 in KS lesion and adjacent tissue were shown by IHC.
(TIF)

Figure S7 Knockdown of *Id1* slightly decreased the proliferation of MM cell. (A) Id1 expression was shown in MM-sh*Ctrl* and MM-sh*Id1* cells by immunoblotting. (B) Knockdown of *Id1* slightly decreased the proliferation of MM cell. Cell proliferation was measured by MTT assay. Data were shown as mean ± s.e.m., n = 3.
(TIF)

Figure S8 Knockdown of *Id2* or *Id3* inhibited the tumorigenicity of KMM cells. (A) Knockdown of *Id2 and Id3* inhibited anchorage-independent growth of KMM cells in soft agar assay. (B, C) Id2 and Id3 expression was detected in KMM-sh*Ctrl*, KMM-sh*Id2* and KMM-sh*Id3* cells by immunoblotting.
(TIF)

Figure S9 Knockdown of either LANA or Smad1 severely impaired the tumorigenicity of KMM cells. (A) Knockdown of *LANA* or *Smad1* dramatically inhibited anchorage-independent cell growth in soft agar assay. (B) Statistic analysis of colonies number in soft agar assays. Data were shown as mean ± s.e.m., n = 3.
(TIF)

Figure S10 Overexpression of Id1 only did not induce MM cell transformation. (A) Overexpression of Id1 did not support anchorage-independent growth of MM cells in soft agar assay (B) Id1 expression was detected in MM-*Id1* and MM cells by immunoblotting. (C) Relative expression of Id1 was shown.
(TIF)

Figure S11 Ectopic expression of Id1 increased the tumorigenecity of KMM cells. (A) Id1 expression was detected in KMM-*Id1* and KMM-*Vector* cells by immunoblotting. Relative expression of Id1 was shown. (B) Ectopic expression of Id1 increased proliferation of KMM cells. Cell proliferation was measured by MTT assay. Data were shown as mean ± s.e.m., n = 3. (C, D) Ectopic expression of Id1 promoted the colony formation ability of KMM cells. Data were shown as mean ± s.e.m., n = 3. * p<0.05. (E, F) Ectopic expression of Id1 promoted anchorage-independent growth of KMM cells. Data were shown as mean ± s.e.m., n = 3. * p<0.05.
(TIF)

Figure S12 Ectopic expression of Id1 significantly rescued Dorsomorphin induced G2/M arrest and cellular toxicity in KMM cells. (A) KMM-*Vector* and KMM-*Id1* cells were treated with DMSO or 5 µM Dorsomorphin for 48 hours. Then the cells were harvested and subjected to PI staining and cell cycle analysis by Mod Fit software. (B) KMM-*Vector* and KMM-*Id1* cells were treated with DMSO or 5 µM Dorsomorphin for 48 hours. Then, the cells were stained with PI solution. The PI subset represented the dead cells.
(TIF)

Figure S13 Ectopic expression of Id1 significantly rescued Dorsomorphin-induced cellular toxicity in 293T cells in a dose-dependent manner. (A) 293T cells were first transfected with 0, 0.5 or 2 µg Id1 for 24 hours, then seeded in 96-well plate and treated with 2.5 µM Dorsomorphin for 48 hours (5 µM). Cell viability was tested by MTT assay. Data were shown as mean ± s.e.m., n = 3. * p<0.05. (B) Expression of Id1 was checked by immunostaining. Relative expression of Id1 was put under the blot.
(TIF)

Figure S14 Expression of Ids was examined in lymphoma cell lines. (A) Expression of Ids was examined in KSHV-positive BCBL1 and BC cells, and in KSHV-negative BJAB cells by immunoblotting. (B) Expression of *Ids* was examined in BJAB and KSHV-BJAB cells by qRT-PCR. Data were shown as mean ± s.e.m., n = 3.
(TIF)
