# Correction: Oncogenic Herpesvirus KSHV Hijacks BMP-Smad1-Id Signaling to Promote Tumorigenesis

**DOI:** 10.1371/journal.ppat.1010390

**Published:** 2022-03-14

**Authors:** Deguang Liang, Hao Hu, Shasha Li, Jiazhen Dong, Xing Wang, Yuhan Wang, Li He, Zhiheng He, Yuan Gao, Shou-Jiang Gao, Ke Lan

An error was made in generating Figure S10 of this article [1]: the GADPH control blot for the experiment shown in Figure 7B was duplicated in the GADPH panel of Figure S10B in error and mislabelled. An updated version of Figure S10 is provided with this Correction. The Id1 and GAPDH blots in panel B have been replaced with the correct data from a different independent replicate carried out as part of the original experiments, and correctly labelled as Id1 and Actin; these errors do not affect the quantitative data reported in panel C. Underlying data supporting the updated figure are in S1, S2, and S3 Files. The authors confirm that Figure 7B in the published article showed the correct data.

The authors apologise for the errors in the published article.

Raw data supporting other results in the published article are available from the corresponding author.

## Supporting information

Figure S10 Overexpression of Id1 only did not induce MM cell transformation. (A) Overexpression of Id1 did not support anchorage-independent growth of MM cells in soft agar assay (B) Id1 expression was detected in MM-*Id1* clones and MM cells by immunoblotting. (C) The relative expression of Id1 in the representative blot in panel B was quantified by densitometric analysis, n = 1.
(TIF)

S1 File Original blot data supporting the Id1 results in the updated Figure S10. (TIF)

S2 File Original blot data supporting the actin results in the updated Figure S10. (TIF)

S3 File Original individual-level quantitative data supporting Figure S10C. (XLSX)
