# Supplementary figures and images for: Oncogenic Herpesvirus KSHV Hijacks BMP-Smad1-Id Signaling to Promote Tumorigenesis
Source: PLoS Pathog. 2014 Jul 10;10(7):e1004253. doi: 10.1371/journal.ppat.1004253 (PMC4092152; doi:10.1371/journal.ppat.1004253)

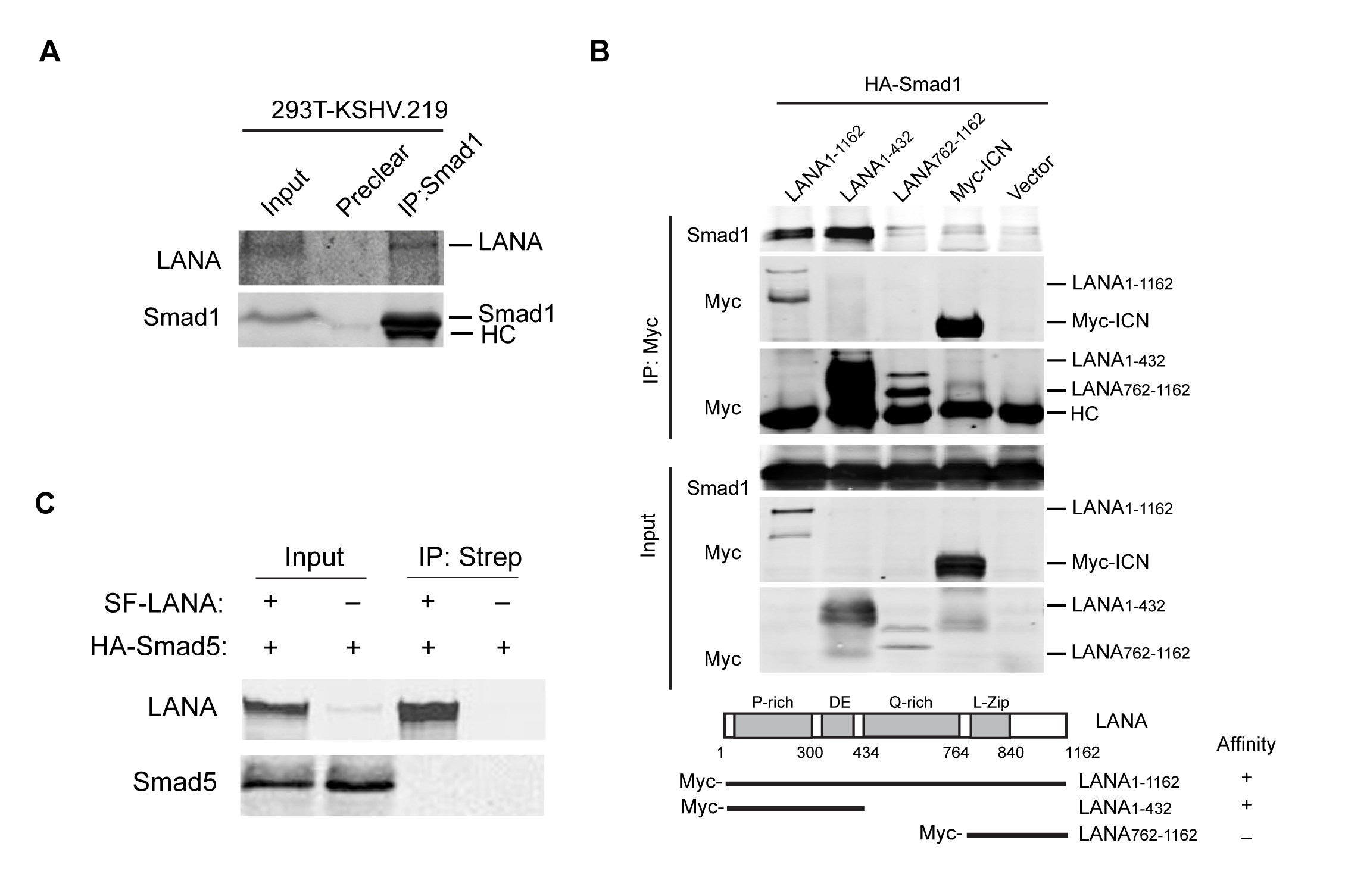

Supplement: Figure S1 — LANA interacted with Smad1 but not Smad5. (A) LANA interacted with Smad1 in KSHV infected cells. 293T-KSHV.219 cells were harvested for endogenous co-IP and immuoblotting as indicated. (B) N-terminal of LANA was responsible for Smad1 binding. HA-Smad1 (12 µg) was co-transfected with different truncated LANA constructs, Intracellular Notch or vector (12 µg each) into 293T cells. Cell lysates were immunoprecipitated as indicated. (C) LANA did not interact with Smad5. HA-Smad5 (12 µg) was co-transfected with SF-LANA or vector (12 µg each) into 293T cells. Cell lysates were immunoprecipitated as indicated. (TIF) [file ppat.1004253.s001.tif]

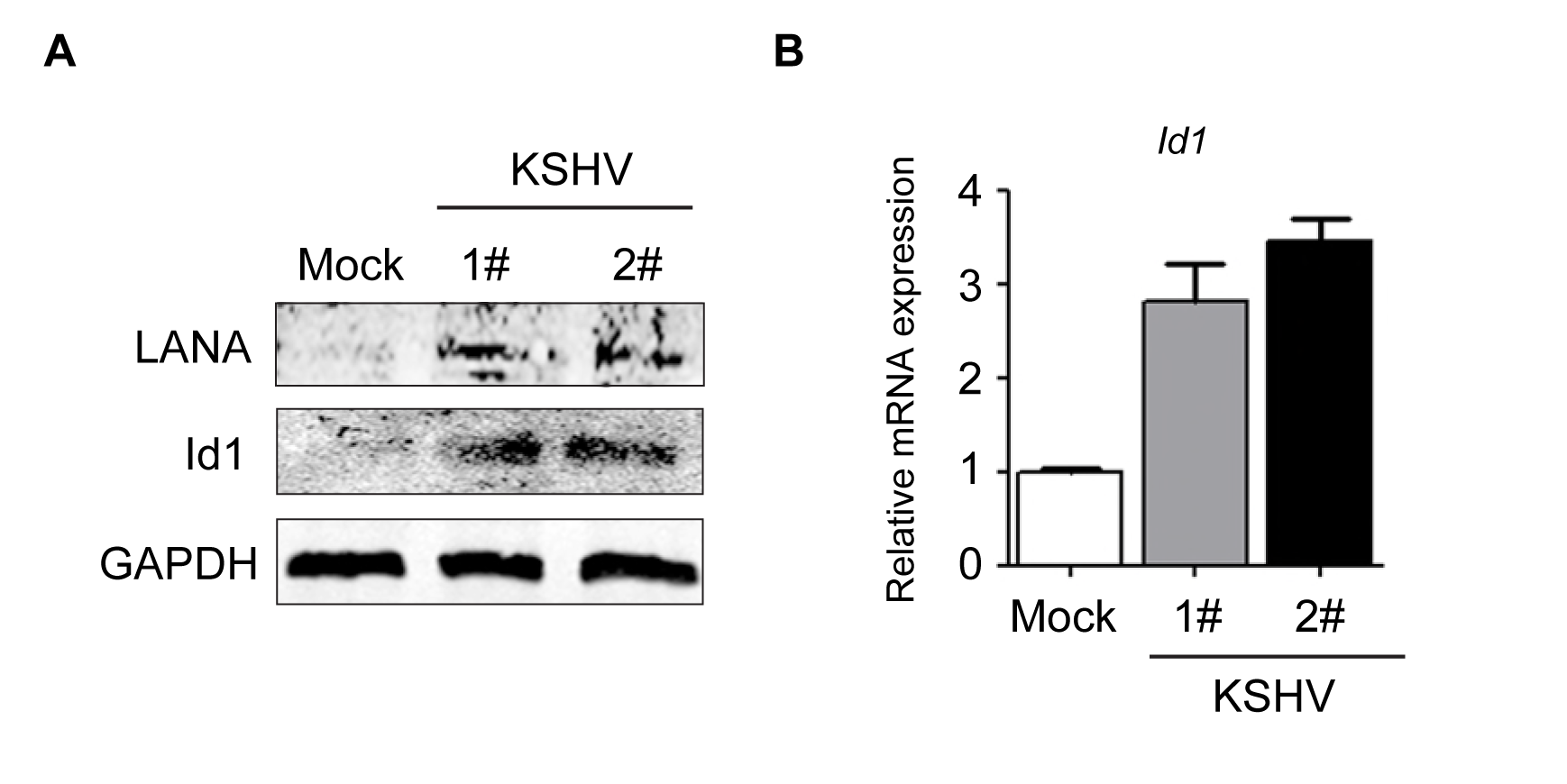

Supplement: Figure S2 — Id1 was up-regulated in KSHV-infected human primary endothelial cells. (A) HUVECs were harvested for immunoblotting as indicated at 24 hours post KSHV infection. (B) HUVECs were harvested for qRT-PCR as indicated at 24 hours post KSHV infection. (TIF) [file ppat.1004253.s002.tif]

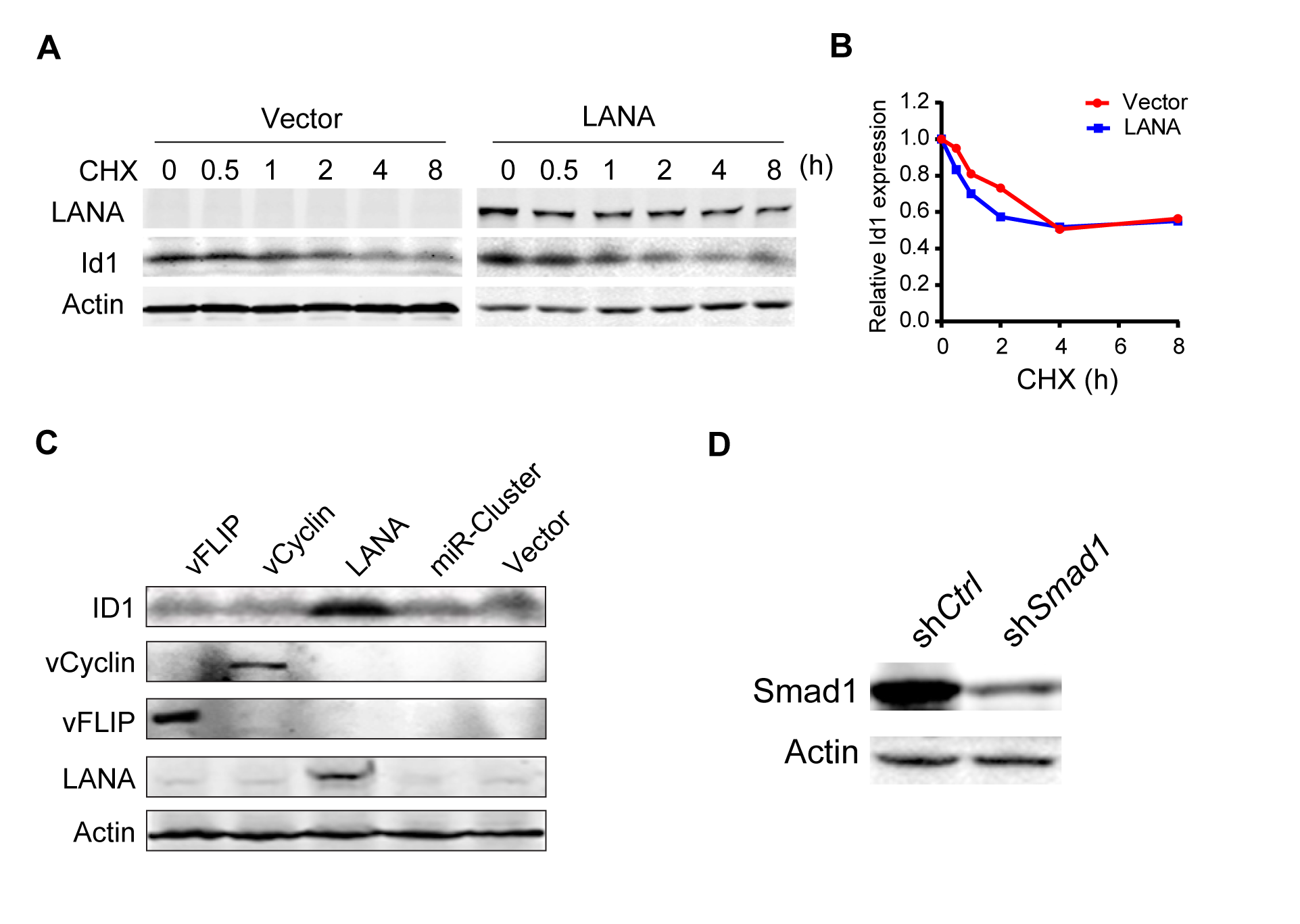

Supplement: Figure S3 — LANA up-regulated Id1 expression in transcription level. (A) LANA did not alter Id1 stability in 293T cells. LANA or vector (12 µg each) transfected 293T cells were treated with 5 µg/ml CHX. Cells were harvested at the indicated times. Cell lysates were analyzed by immunoblotting. (B) Relative expression of Id1 after CHX treatment was quantified. (C) LANA but no other latent genes were responsible for Id1 up-regulation. vFLIP, vCyclin, LANA, miR-Cluster or Vector (12 µg each) were transfected into 293T cells. Cell lysates were analyzed by immunoblotting. (D) Expression of Smad1 in 293T-shSmad1 and 293T-shCtrl cells was detected by immunoblotting. (TIF) [file ppat.1004253.s003.tif]

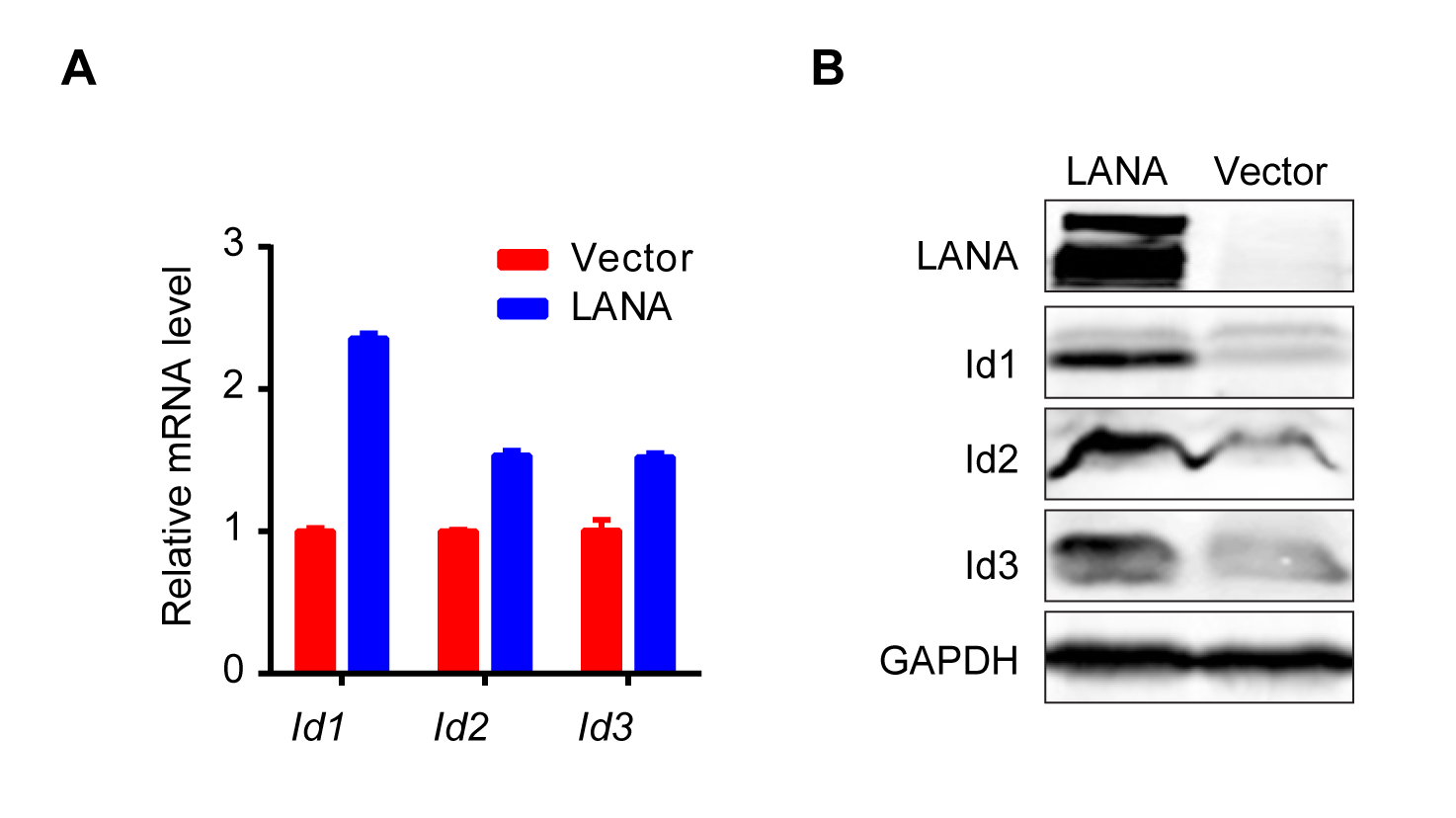

Supplement: Figure S4 — Ids were up-regulated in LANA transfected 293T cells in both mRNA level (A) and protein level (B). (TIF) [file ppat.1004253.s004.tif]

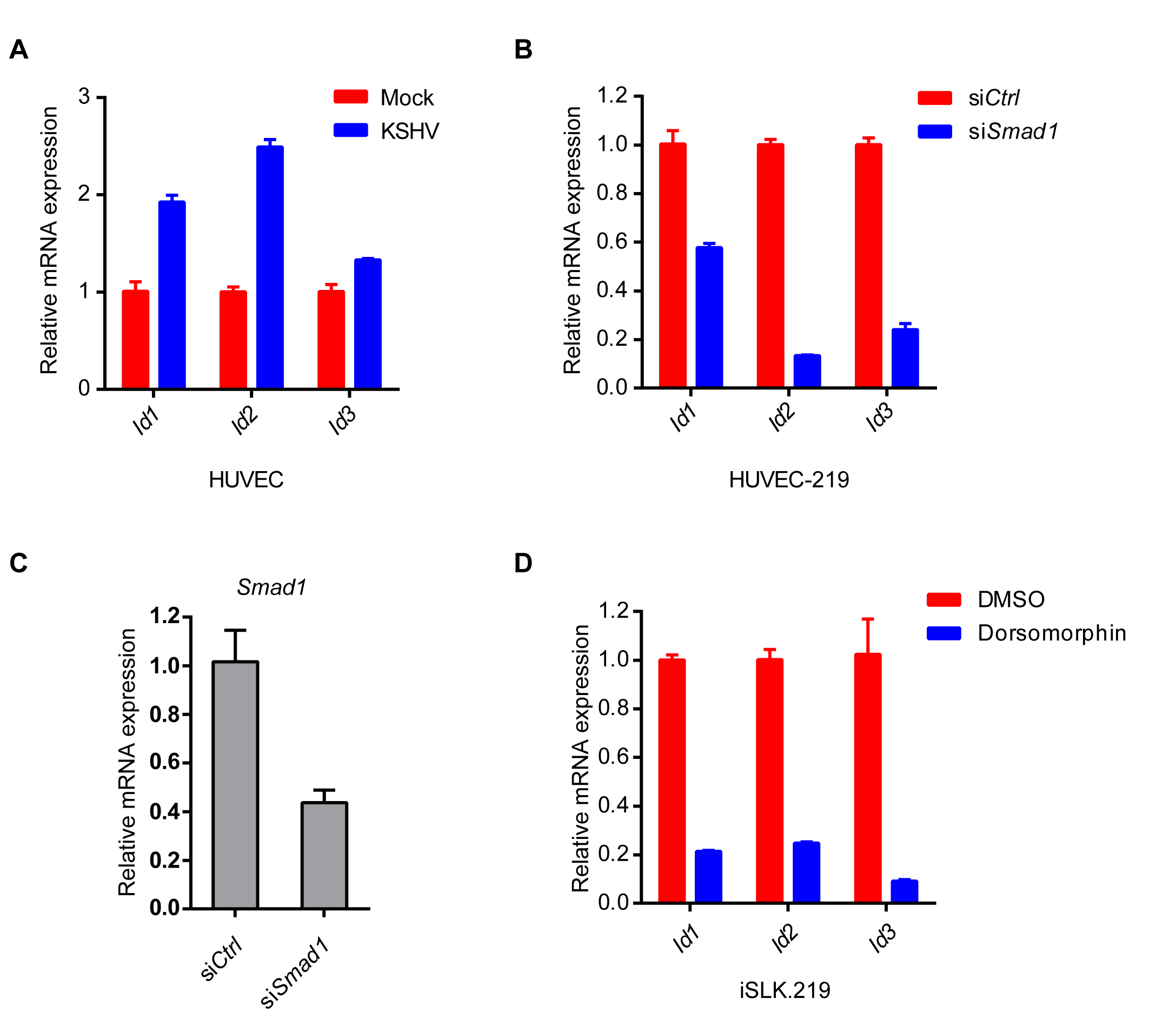

Supplement: Figure S5 — Ids were generally up-regulated in KSHV infected cells through BMP-Smad1 signaling pathway. (A) Expression of Ids was up-regulated in KSHV infected HUVECs. (B) Knockdown of Smad1 significantly impaired the expression of Id1, Id2 and Id3 in KSHV infected HUVECs. (C) Knockdown efficiency of siSmad1 was checked by qRT-PCR. (D) Dorsomorphin dramatically repressed Id1, Id2 and Id3 in iSLK.219 cells. (TIF) [file ppat.1004253.s005.tif]

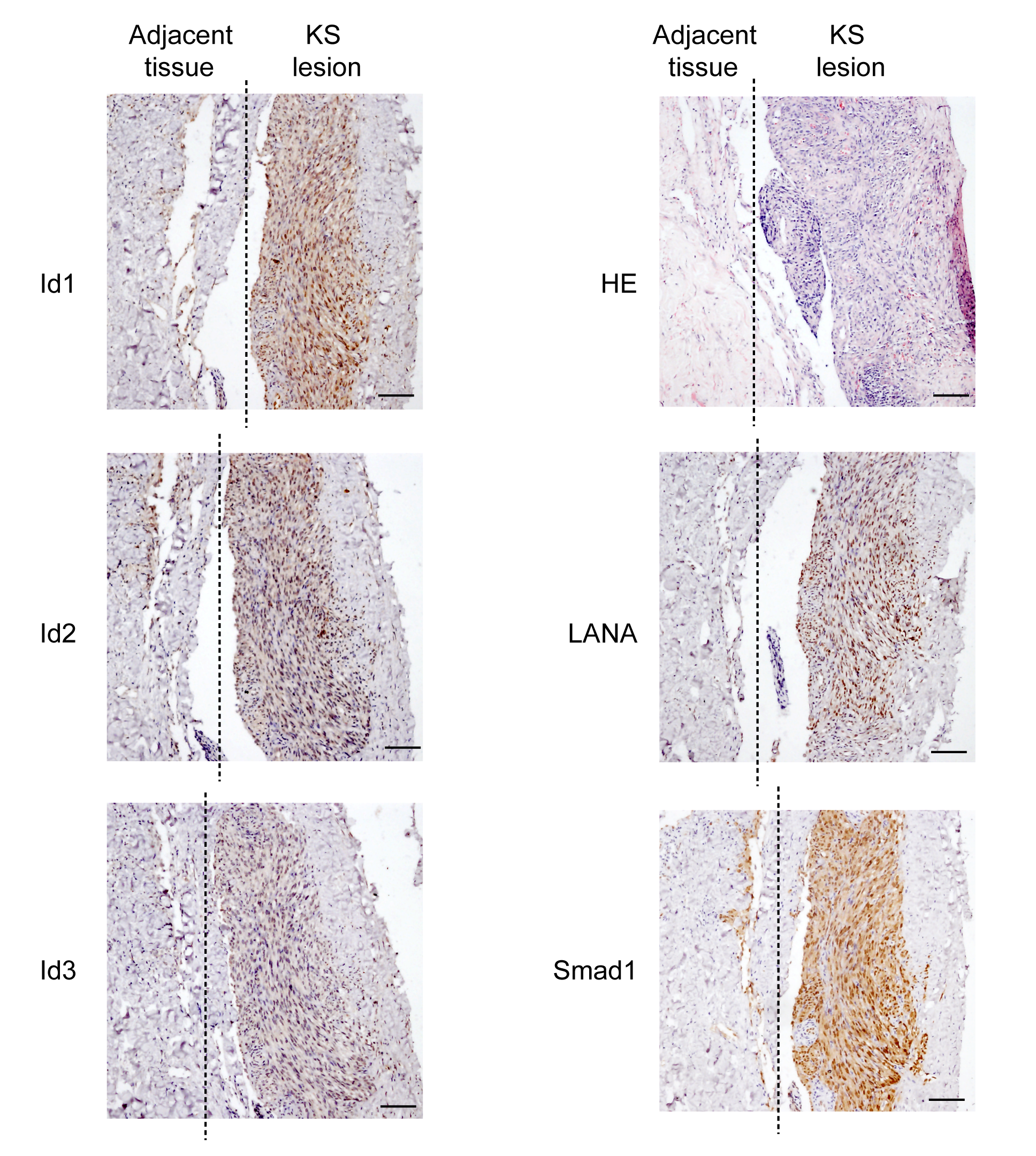

Supplement: Figure S6 — Expression of Ids, LANA and Smad1 in KS lesion and adjacent tissue were shown by IHC. (TIF) [file ppat.1004253.s006.tif]

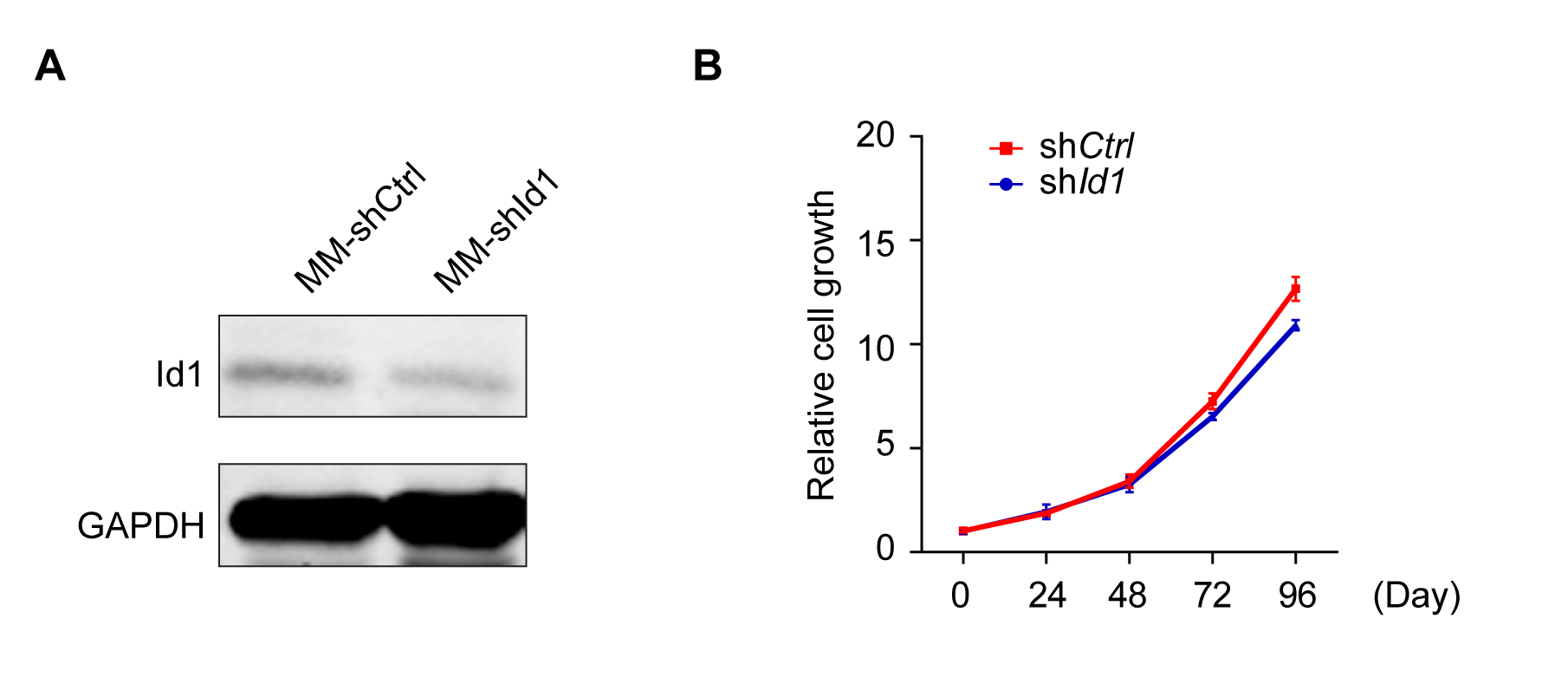

Supplement: Figure S7 — Knockdown of Id1 slightly decreased the proliferation of MM cell. (A) Id1 expression was shown in MM-shCtrl and MM-shId1 cells by immunoblotting. (B) Knockdown of Id1 slightly decreased the proliferation of MM cell. Cell proliferation was measured by MTT assay. Data were shown as mean ± s.e.m., n = 3. (TIF) [file ppat.1004253.s007.tif]

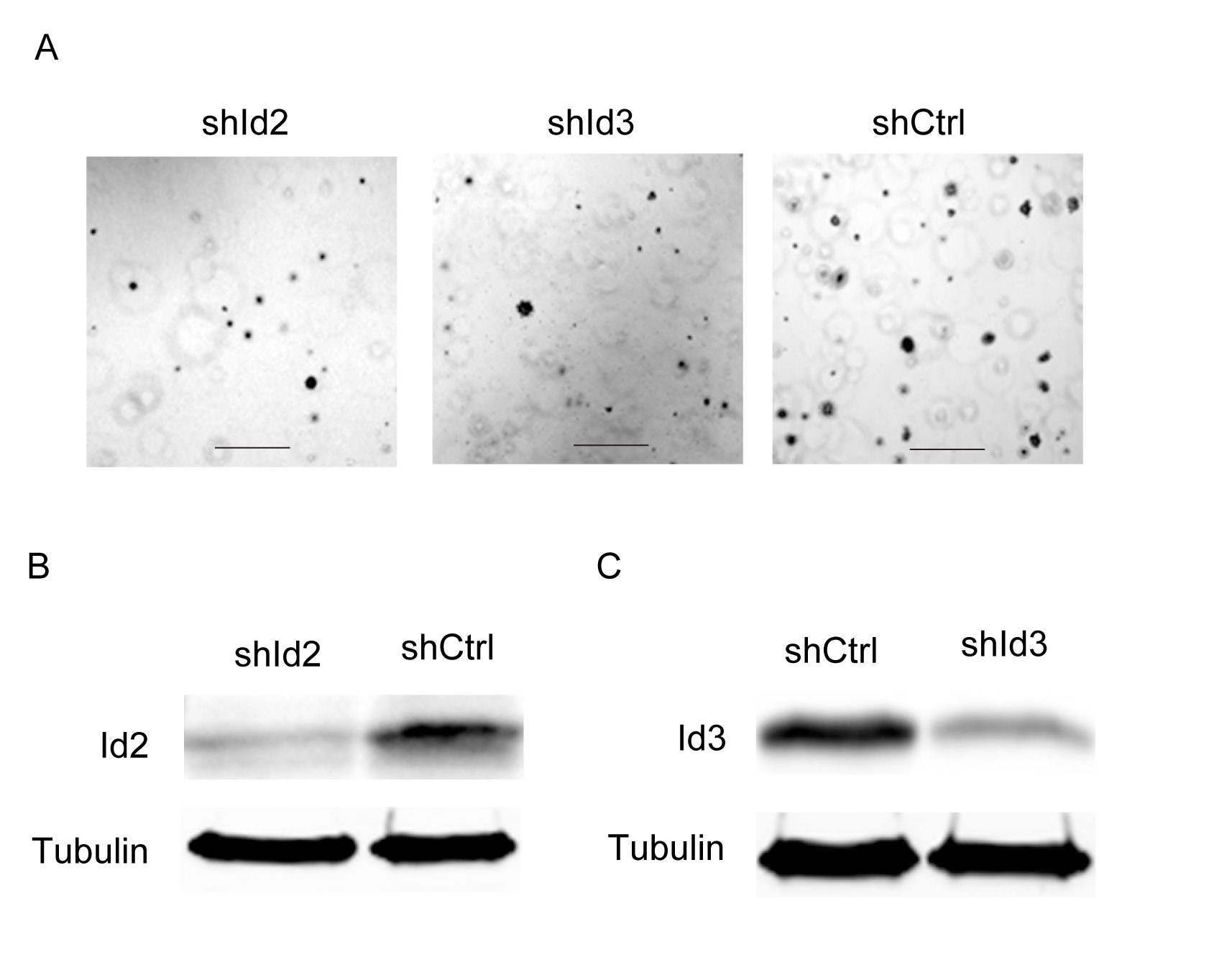

Supplement: Figure S8 — Knockdown of Id2 or Id3 inhibited the tumorigenicity of KMM cells. (A) Knockdown of Id2 and Id3 inhibited anchorage-independent growth of KMM cells in soft agar assay. (B, C) Id2 and Id3 expression was detected in KMM-shCtrl, KMM-shId2 and KMM-shId3 cells by immunoblotting. (TIF) [file ppat.1004253.s008.tif]

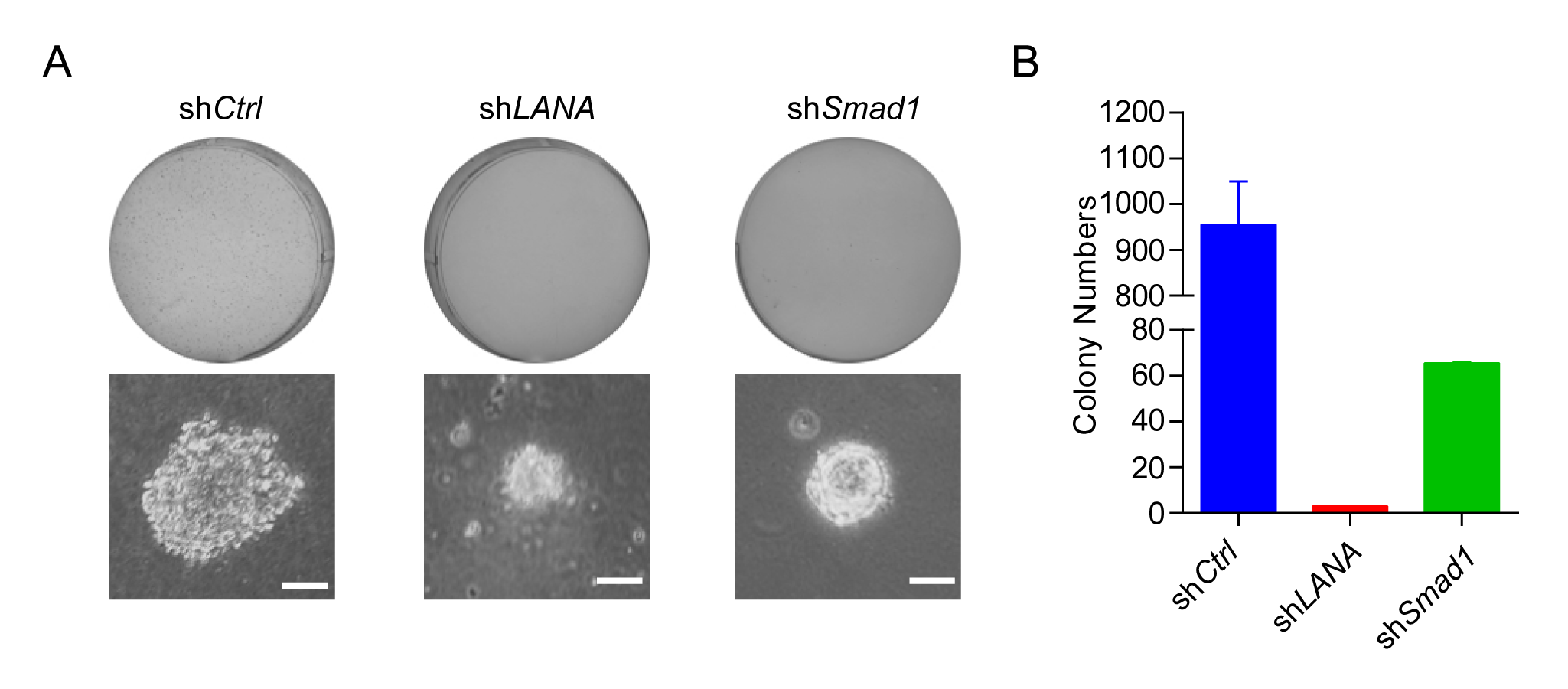

Supplement: Figure S9 — Knockdown of either LANA or Smad1 severely impaired the tumorigenicity of KMM cells. (A) Knockdown of LANA or Smad1 dramatically inhibited anchorage-independent cell growth in soft agar assay. (B) Statistic analysis of colonies number in soft agar assays. Data were shown as mean ± s.e.m., n = 3. (TIF) [file ppat.1004253.s009.tif]

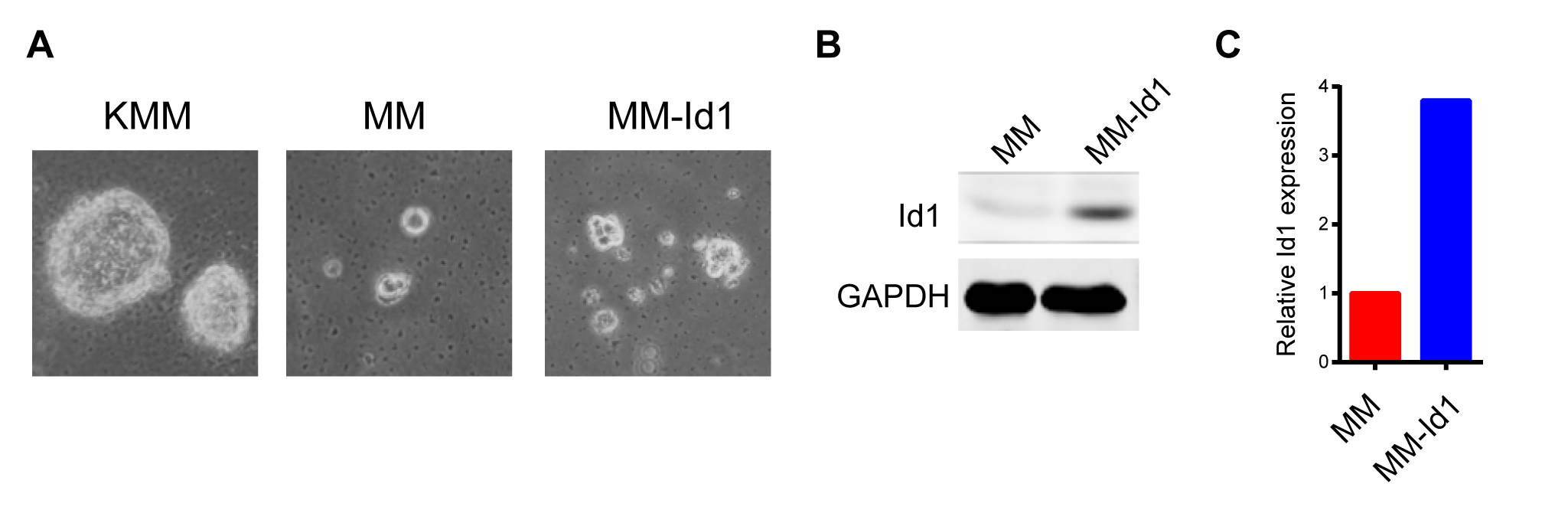

Supplement: Figure S10 — Overexpression of Id1 only did not induce MM cell transformation. (A) Overexpression of Id1 did not support anchorage-independent growth of MM cells in soft agar assay (B) Id1 expression was detected in MM-Id1 and MM cells by immunoblotting. (C) Relative expression of Id1 was shown. (TIF) [file ppat.1004253.s010.tif]

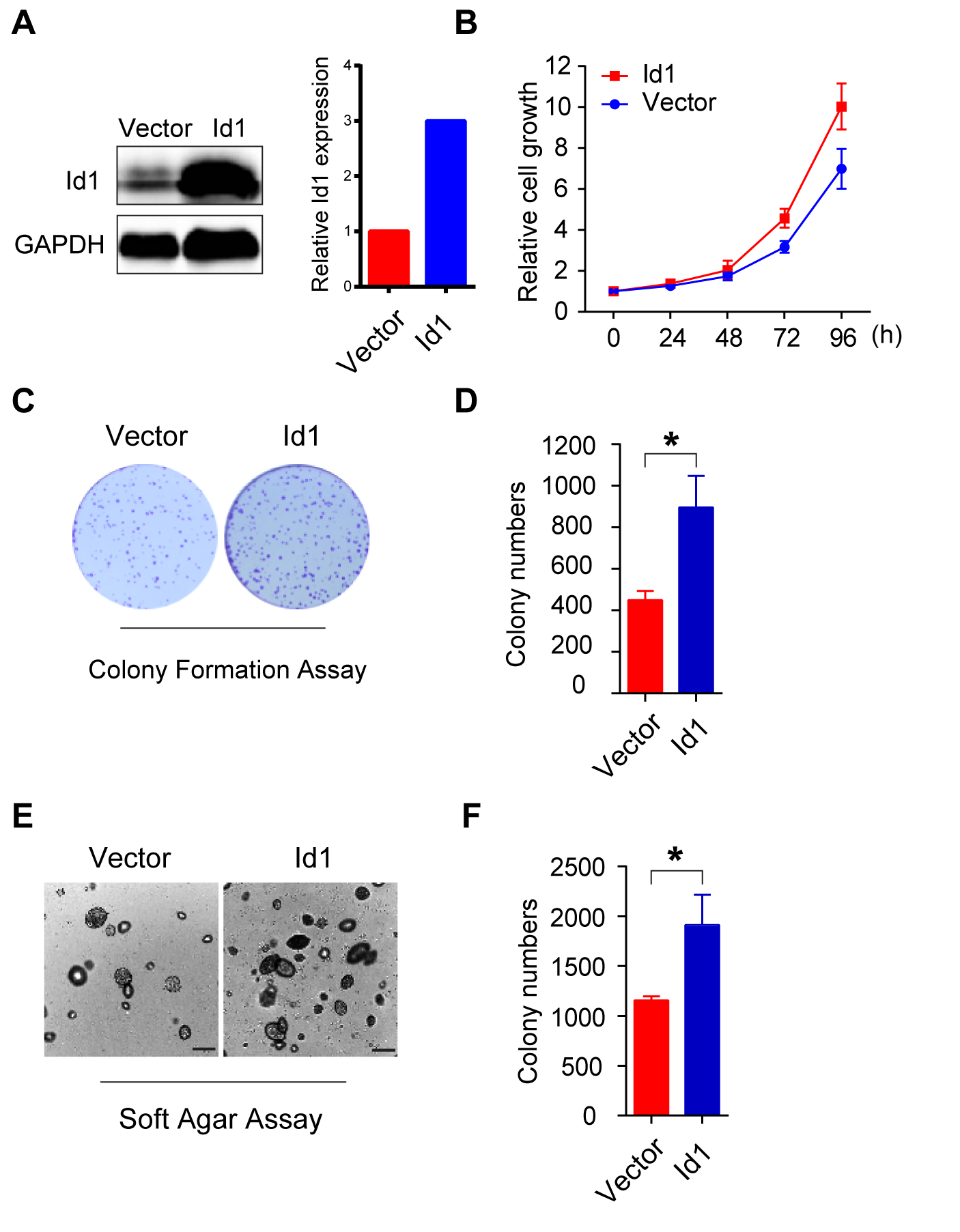

Supplement: Figure S11 — Ectopic expression of Id1 increased the tumorigenecity of KMM cells. (A) Id1 expression was detected in KMM-Id1 and KMM-Vector cells by immunoblotting. Relative expression of Id1 was shown. (B) Ectopic expression of Id1 increased proliferation of KMM cells. Cell proliferation was measured by MTT assay. Data were shown as mean ± s.e.m., n = 3. (C, D) Ectopic expression of Id1 promoted the colony formation ability of KMM cells. Data were shown as mean ± s.e.m., n = 3. * p<0.05. (E, F) Ectopic expression of Id1 promoted anchorage-independent growth of KMM cells. Data were shown as mean ± s.e.m., n = 3. * p<0.05. (TIF) [file ppat.1004253.s011.tif]

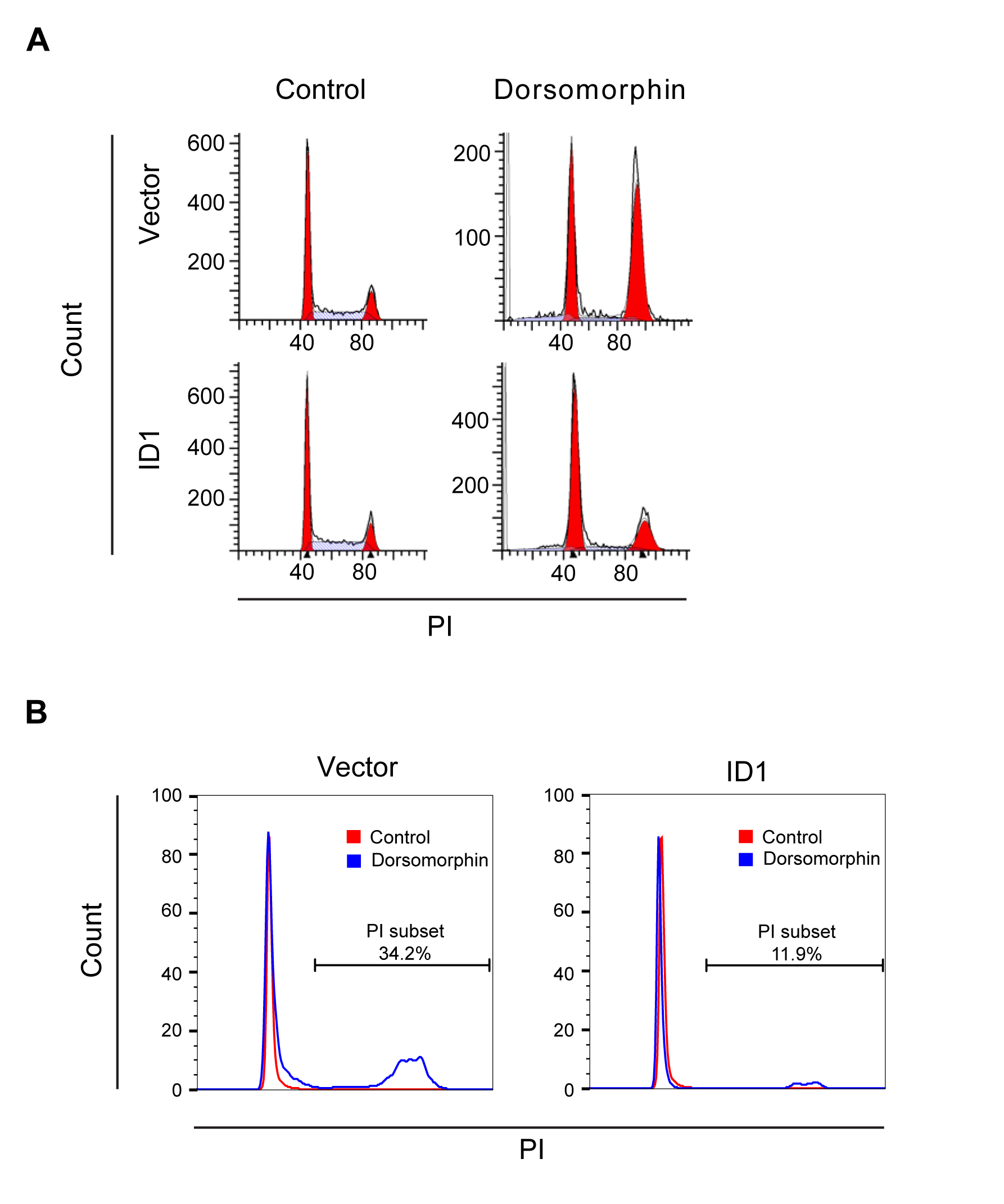

Supplement: Figure S12 — Ectopic expression of Id1 significantly rescued Dorsomorphin induced G2/M arrest and cellular toxicity in KMM cells. (A) KMM-Vector and KMM-Id1 cells were treated with DMSO or 5 µM Dorsomorphin for 48 hours. Then the cells were harvested and subjected to PI staining and cell cycle analysis by Mod Fit software. (B) KMM-Vector and KMM-Id1 cells were treated with DMSO or 5 µM Dorsomorphin for 48 hours. Then, the cells were stained with PI solution. The PI subset represented the dead cells. (TIF) [file ppat.1004253.s012.tif]

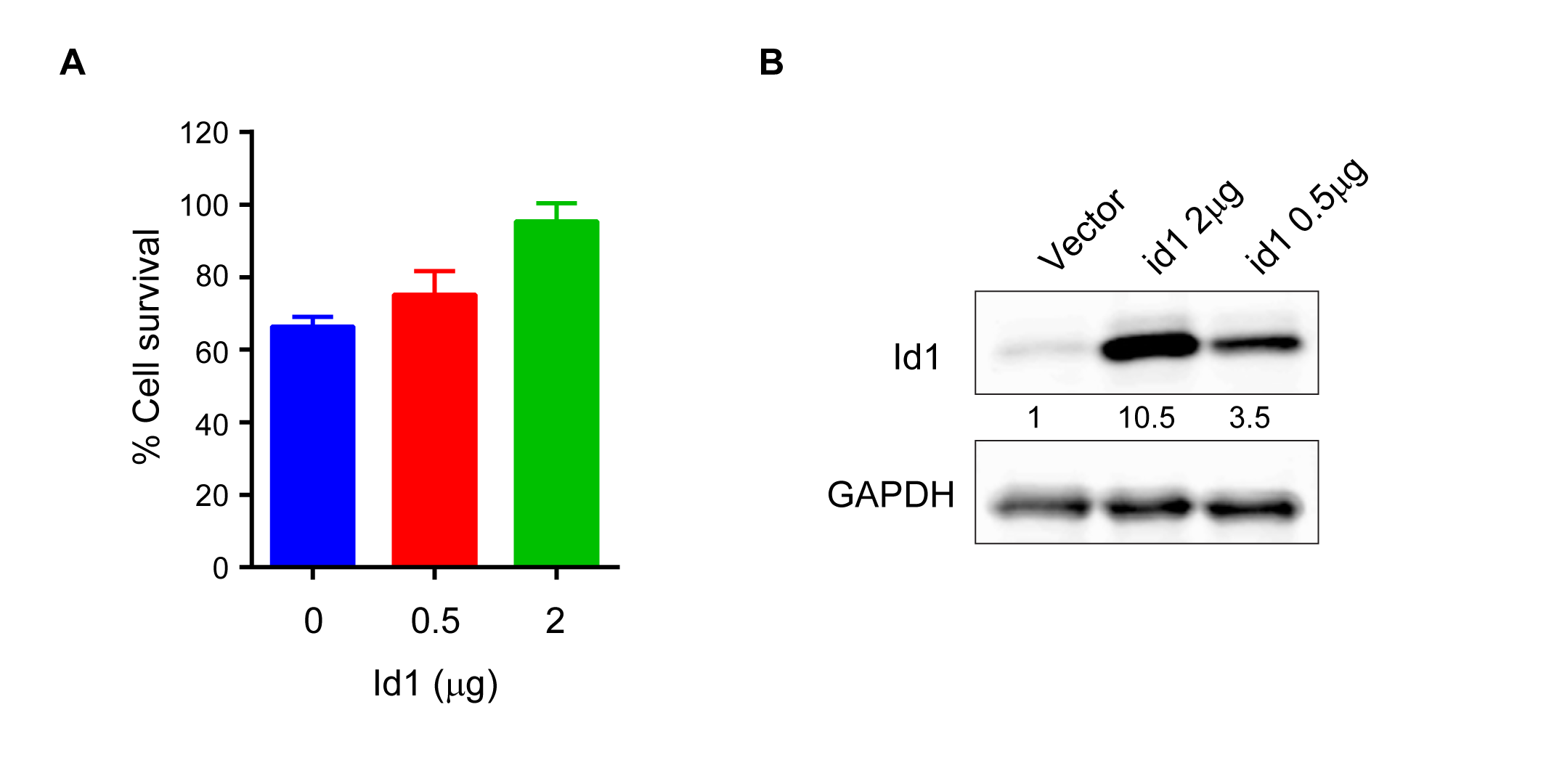

Supplement: Figure S13 — Ectopic expression of Id1 significantly rescued Dorsomorphin-induced cellular toxicity in 293T cells in a dose-dependent manner. (A) 293T cells were first transfected with 0, 0.5 or 2 µg Id1 for 24 hours, then seeded in 96-well plate and treated with 2.5 µM Dorsomorphin for 48 hours (5 µM). Cell viability was tested by MTT assay. Data were shown as mean ± s.e.m., n = 3. * p<0.05. (B) Expression of Id1 was checked by immunostaining. Relative expression of Id1 was put under the blot. (TIF) [file ppat.1004253.s013.tif]

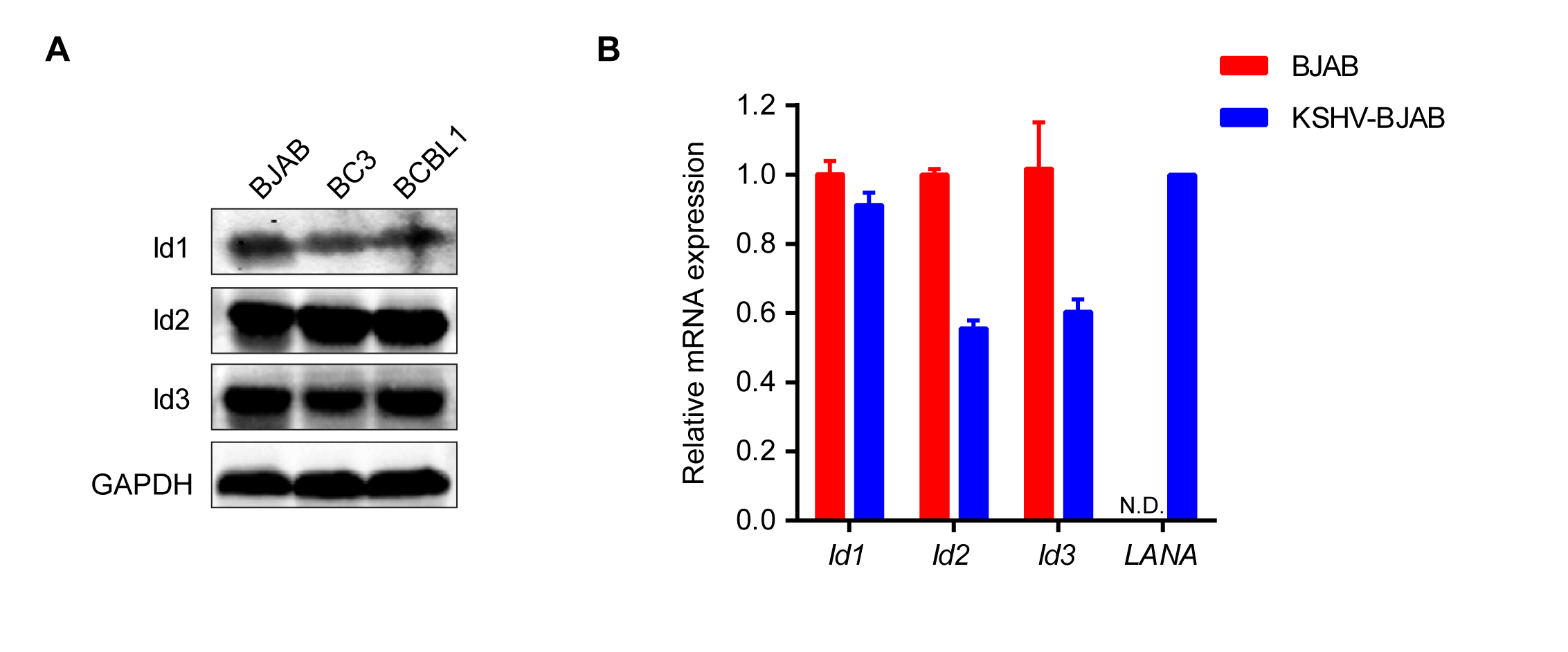

Supplement: Figure S14 — Expression of Ids was examined in lymphoma cell lines. (A) Expression of Ids was examined in KSHV-positive BCBL1 and BC cells, and in KSHV-negative BJAB cells by immunoblotting. (B) Expression of Ids was examined in BJAB and KSHV-BJAB cells by qRT-PCR. Data were shown as mean ± s.e.m., n = 3. (TIF) [file ppat.1004253.s014.tif]
