# Supplementary figures and images for: Correction: Oncogenic Herpesvirus KSHV Hijacks BMP-Smad1-Id Signaling to Promote Tumorigenesis
Source: PLoS Pathog. 2022 Mar 14;18(3):e1010390. doi: 10.1371/journal.ppat.1010390 (PMC8920289; doi:10.1371/journal.ppat.1010390)

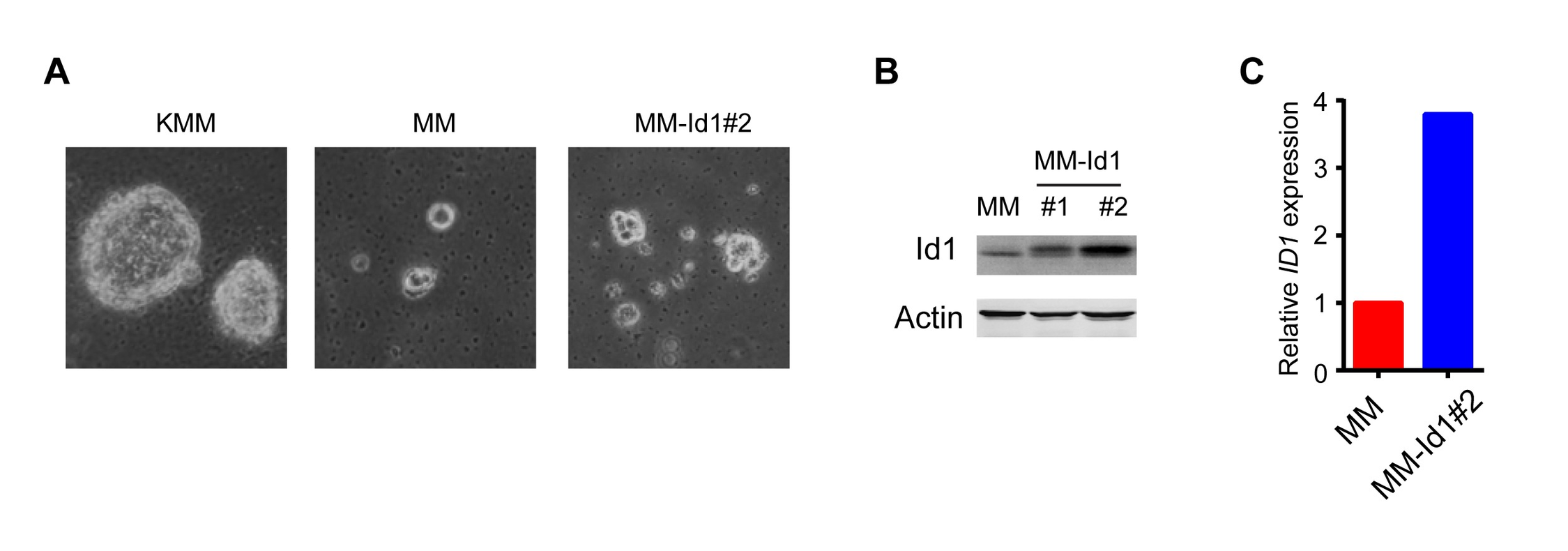

Supplement: Figure S10 — (A) Overexpression of Id1 did not support anchorage-independent growth of MM cells in soft agar assay (B) Id1 expression was detected in MM-Id1 clones and MM cells by immunoblotting. (C) The relative expression of Id1 in the representative blot in panel B was quantified by densitometric analysis, n = 1. (TIF) [file ppat.1010390.s001.tif]

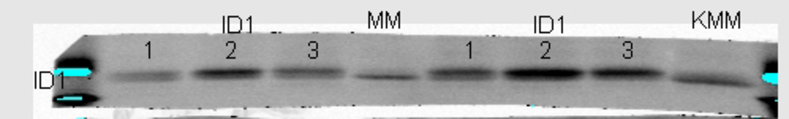

Supplement: S1 File — (TIF) [file ppat.1010390.s002.tif]

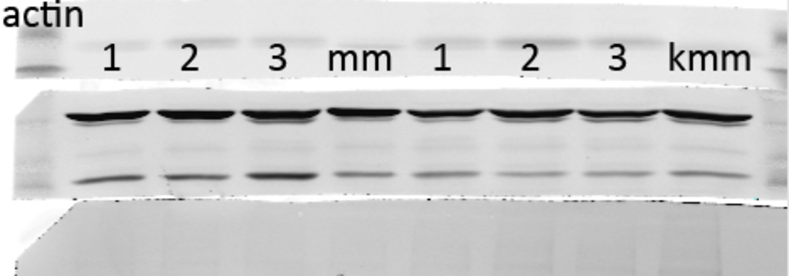

Supplement: S2 File — (TIF) [file ppat.1010390.s003.tif]
